# Deciphering the atomic-scale structural origin for large dynamic electromechanical response in lead-free Bi_0.5_Na_0.5_TiO_3_-based relaxor ferroelectrics

**DOI:** 10.1038/s41467-022-34062-6

**Published:** 2022-10-25

**Authors:** Jie Yin, Xiaoming Shi, Hong Tao, Zhi Tan, Xiang Lv, Xiangdong Ding, Jun Sun, Yang Zhang, Xingmin Zhang, Kui Yao, Jianguo Zhu, Houbing Huang, Haijun Wu, Shujun Zhang, Jiagang Wu

**Affiliations:** 1 Department of Materials Science, Sichuan University, Chengdu, China; 2 State Key Laboratory for Mechanical Behavior of Materials, Xi’an Jiaotong University, Xi’an, China; 3 Advanced Research Institute of Multidisciplinary Science, Beijing Institute of Technology, Beijing, China; 4 Physics Department, Southwest Minzu University, Chengdu, China; 5 Instrumental Analysis Center of Xi’an Jiaotong University, Xi’an Jiaotong University, 710049 Xi’an, China; 6 Shanghai Synchrotron Radiation Facility, Shanghai Institute of Applied Physics, Chinese Academy of Sciences, Pudong New Area, Shanghai, China; 7 Institute of Materials Research and Engineering, Agency for Science, Technology and Research (A*STAR), Singapore, 138634 Singapore; 8 Institute for Superconducting and Electronic Materials, Australian Institute of Innovative Materials, University of Wollongong, Wollongong, NSW Australia

**Keywords:** Electronic properties and materials, Ferroelectrics and multiferroics

## Abstract

Despite the extraordinary electromechanical properties of relaxor ferroelectrics, correlating their properties to underlying atomic-scale structures remains a decisive challenge for these “mess” systems. Here, taking the lead-free relaxor ferroelectric Bi_0.5_Na_0.5_TiO_3_-based system as an example, we decipher the atomic-scale structure and its relationship to the polar structure evolution and large dynamic electromechanical response, using the direct atomic-scale point-by-point correlation analysis. With judicious chemical modification, we demonstrate the increased defect concentration is the main driving force for deviating polarizations with high-angle walls, leading to the increased random field. Meanwhile, the main driving force for deviating polarizations with low-angle walls changes from the anti-phase oxygen octahedral tilting to the multidirectional A-O displacement, leading to the decreased anisotropy field. Benefiting from the competitive and synergetic equilibrium of anisotropic field versus random field, the facilitated polarization rotation and extension versus facilitated domain switching are identified to be responsible for the giant electromechanical response. These observations lay a foundation for understanding the “composition-structure-property” relationships in relaxor ferroelectric systems, guiding the design of functional materials for electromechanical applications.

## Abstract (web summary)

For relaxor ferroelectrics, correlating their properties and local structures is challenging. Here, the authors correlate the atomic-scale structure and the large dynamic electromechanical property in Bi_0.5_Na_0.5_TiO_3_-based relaxor ferroelectrics.

## Introduction

**Fig. 1:**
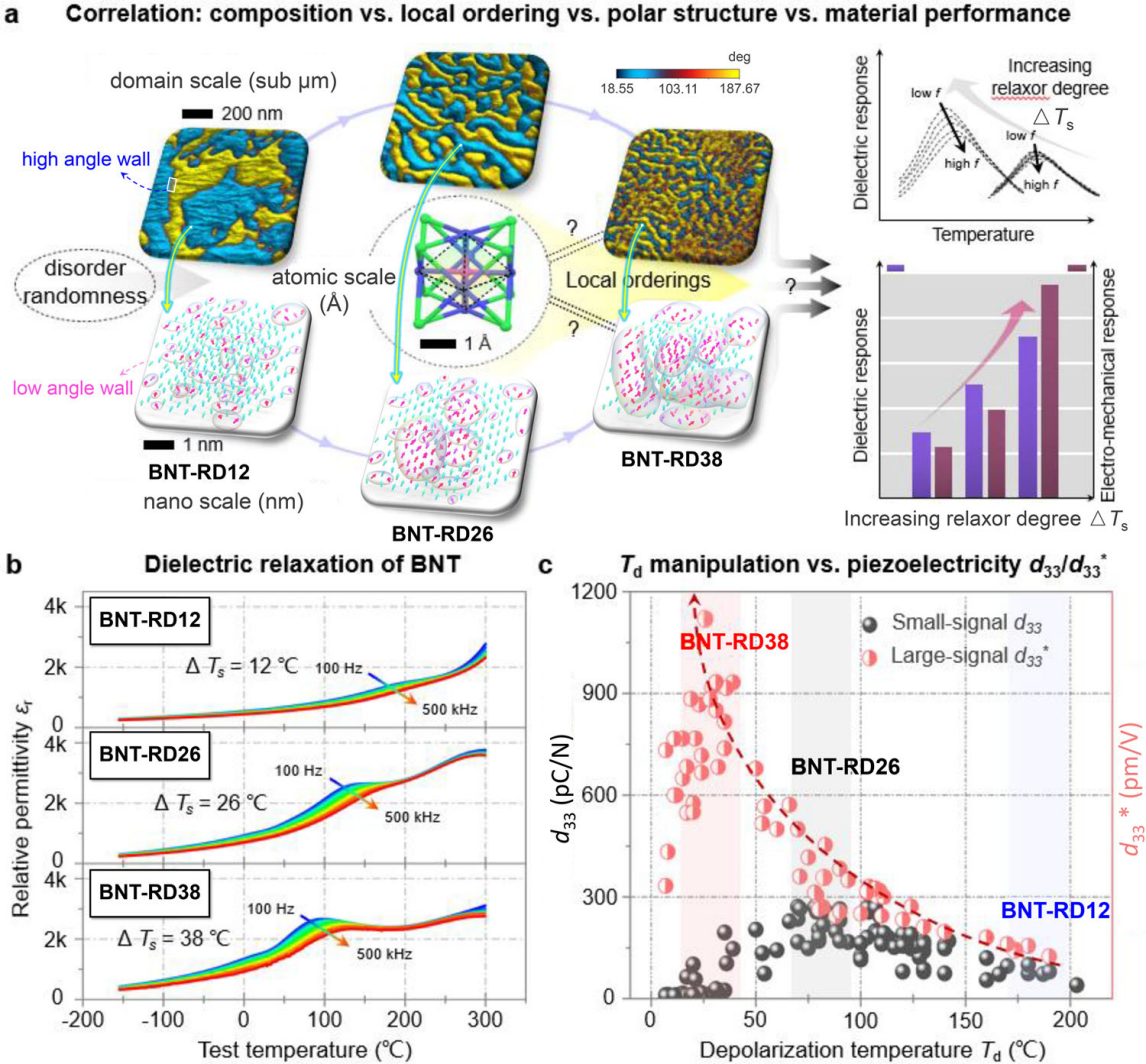
Challenge in correlating local structures with dielectric and electromechanical properties in relaxor ferroelectrics. **a** Schematic illustration of introducing local disorder and randomness to enhance the dielectric and electromechanical responses in ferroelectric oxides. Although the improved properties are believed to originate from the modulated polar structures with deviated polarizations, the underlying nature of these disordered structures remains controversial. Domain structures are obtained by piezoelectric-force microscopy (PFM) on BNT-RD12, BNT-RD26, BNT-RD38 compositions. **b** Dielectric relaxor behaviors of selected BNT-RD12, BNT-RD26, BNT-RD38 compositions. △*T*_s_ is used to evaluate the relaxor degree, which is the shift of the permittivity peak from 100 Hz to 500 kHz. **c** Compilation of the electromechanical properties (*d*_33_ and *d*_33_^*^) against depolarization temperature (*T*_d_) for the BNT-based system. See data source in Supplementary References.

Building ordered lattice is fundamental to the science of condensed matter physics, which defines the formation of metals, insulators, semiconductors, superconductors, and the structure of matter itself^1^. By introducing local disorder and randomness, the phenomenon of deviations from the perfection occurring at the nanoscale, breaks the long-range correlation and average symmetry, providing enormous freedoms for modifying the properties of matter^2^. This strategy has been successfully established in perovskite relaxor ferroelectrics—complex oxides exhibiting the frequency-dependent diffusive phase transition that can substantially enhance dielectric and electromechanical properties^3–5^. Although these improved properties are often attributed to local (nanoscale) symmetry breaking, due to the lack of a clear understanding of their underlying structures, correlating the microscopic physical nature to material performance remains a decisive challenge in these “mess” systems (Fig. 1a)^6–9^.

Growing environmental concerns over lead toxicity have driven the search for lead-free alternatives in recent years^5,10^. Inspired by the heterovalent B-site cations (Mg^2+^, Nb^5+^) in the canonical relaxor system PbMg_1/3_Nb_2/3_O_3_ (PMN), lead-free Bi_0.5_Na_0.5_TiO_3_ (BNT)-based relaxor systems with heterovalent A-site cations (Bi^3+^, Na^+^) are expected to achieve comparable electromechanical properties that have been reported in Pb-based counterparts^11^. However, PMN- and BNT-based relaxors possess different atomic displacement due to the different interactions between cations and anions, thus have different local structures, leading to distinct electromechanical properties^12,13^, even though they exhibit similar frequency-dependent diffused dielectric relaxation behaviors (Fig. 1b). The representative example is the small electric field-induced high piezoelectric coefficient *d*_33_ in PMN-PT ceramic system^3^, while BNT-based ceramics exhibit considerable large electric field-induced strain, as reflected by *d*_33_^*^ (*S*_max_/*E*_max_)^14^. After two decades of extensive research, by introducing local disorder and randomness into BNT-based relaxor systems, *d*_33_^*^ has reached comparable or even higher values to that of lead-based counterparts (*d*_33_^*^ > 1000 pm/V, see Fig. 1c). However, apart from the polar-nano-regions (PNRs) model in lead-based systems^13^, the microscopic nature has not been clearly captured in BNT-based relaxor systems. Therefore, a complete composition–structure–property correlation has not been established, which restricts the advancement of lead-free relaxor systems.

Here, we use atomic-scale correlation analysis in BNT-based lead-free relaxor systems, to decipher the local orderings and their contributions to the polar structure and relaxor ferroelectric properties, especially for the observed giant electromechanical property. This work is motivated by recent investigations on the performance modification of BNT-based relaxor systems^10,15,16^, where the composition–structure–performance correlation is still not resolved. In the BNT-based system, electromechanical properties and relaxor degrees vary with composition, allowing clarification of the composition–structure-performance correlation in selected compositions. In this research, compositions with different relaxor degrees were selected, as marked by BNT-RD12 (△*T*_s_ = 12 °C), BNT-RD26 (△*T*_s_ = 26 °C), BNT-RD38 (△*T*_s_ = 38 °C). BNT-RD12 is for pure BNT matrix, BNT-RD26 is for A-site-modified BNT-based system, while BNT-RD38 is for an additional B-site-modified system based on BNT-RD26 (see details of the selected compositions in “Methods”). With the data acquired based on the aberration-corrected scanning transmission electron microscopy (STEM) [high-angle annular dark-field (HAADF) and annular bright-field (ABF)], the real-spatial anion/cation displacement behaviors together with the nanoscale polar structure, and their underlying correlation to chemical/structural orderings are revealed^7^. Combining the atomic-scale STEM, electric field (*E*-field)-dependent synchrotron X-ray diffraction and dynamic property measurements, first-principles calculation and phase-field simulation are used to establish the composition-structure-performance correlation in a BNT-based relaxor system.

## Results

### Atomic-scale polarization variation and cation/anion displacement behavior

The ground state of the polar-nano regions (PNRs) in relaxor ferroelectric systems has been a long-standing issue^6–8^. Here, to reveal the structural origin, we explore the atomic displacement and local polarization of the BNT-based system. For each of the studied samples, projected positions of cations and anions are detected across three different locations, and the detail is provided in “Methods”. These coordinate data are then used to reconstruct the underlying polar structures and reveal the atomic displacement behaviors. The projected polarization vectors are evaluated based on calculating the centroid difference between cations and anions (see Supplementary Methods 1.3.3 and Supplementary Figs. 1 and 2)^7,17^. It is interesting to note that within a large-size domain separated by high-angle domain walls, the smaller-size polar structures connect with each other smoothly.

**Fig. 2:**
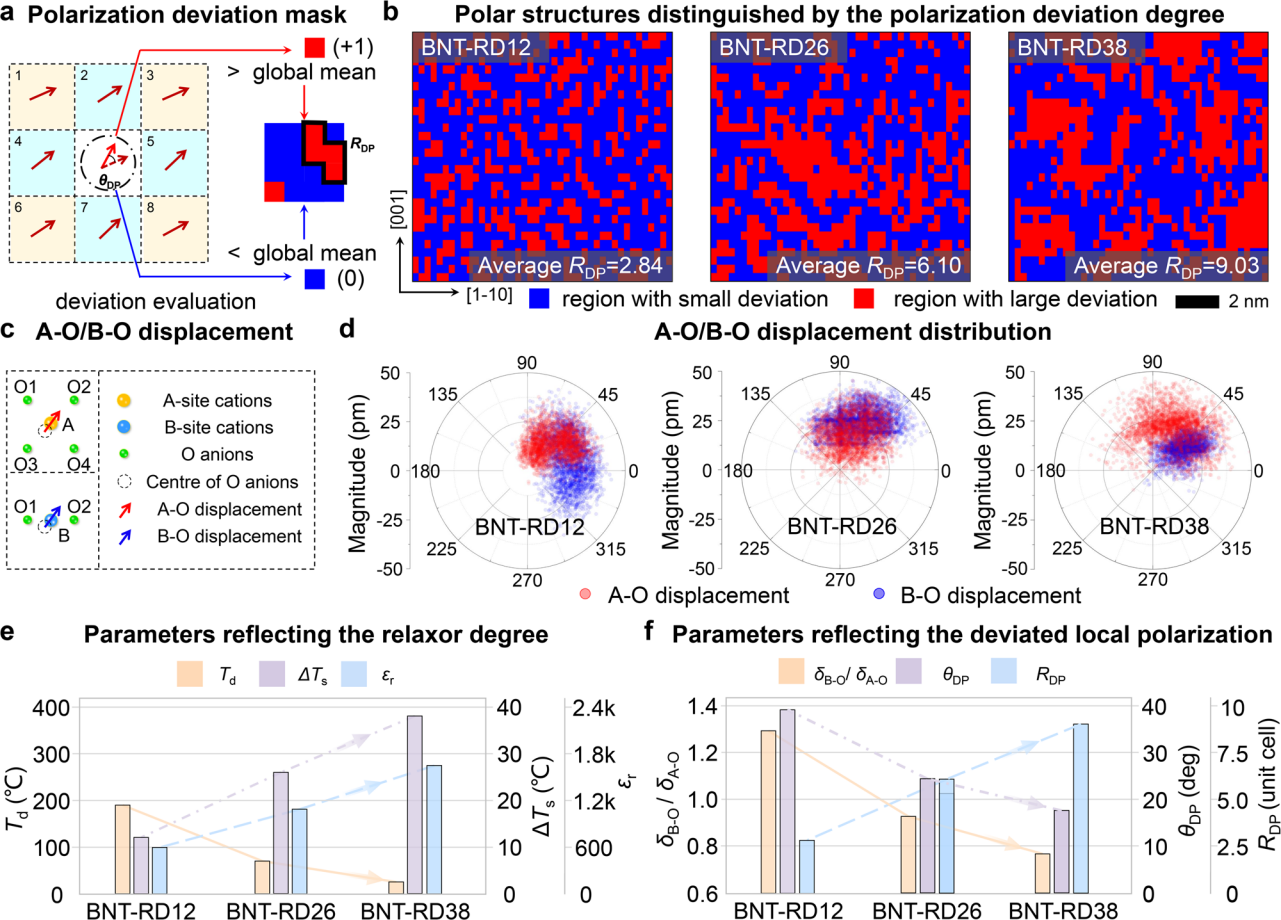
Structural mapping on ionic displacement and polarization variation. **a** Schematic illustration of the method applied to evaluate the deviation degree of polarizations. **b** The [110]_p_ projected local structure reflecting different deviation degrees of polarization angles in the BNT-based system, according to the evaluation principle in (**a**). **c** Schematic illustration of A–O/B–O displacement calculated by the relative offset of the A/B-site cations from the center of four/two adjacent oxygen anions. **d** Compilation of the relative A–O/B–O displacement data in polar plots, which delineate the orientation and magnitude of displacement between cations and anions. **e**, **f** Comparison among the parameters reflecting (**e**) the increasing relaxor degree and (**f**) the deviated local polarization in the BNT-based system.

To elucidate the local polarization evolution in the BNT-based system, the deviation degree of polarization was evaluated. For each unit cell, the deviated angle of polarization (*θ*_DP_) is defined as *θ*_DP_ = ∑_i_|*θ*_i_ − *θ*_0_|/8, where *θ*_0_ is the polarization angle of the central unit cell and *θ*_i_ is the polarization angle of the unit cells surrounding the central unit cell (Fig. 2a)^7^. After applying a global median threshold on *θ*_DP_ (see Supplementary Notes 3.1), these polar regions can be differentiated into two parts with a large/small degree of deviation (Fig. 2b)^7,17^. The red regions, where the angle of the polarization vectors exhibits the larger deviation, connect the smoothly changing polar structures (blue structures). These results present an obvious structure transition in different compositions, which can be reflected by the size of the red regions (*R*_DP_, the number of unit cells for each closed red region). That is, as the doping concentration increases, the dispersed small-size red regions tend to grow into large-size ones (insets of Fig. 2b provide the average *R*_DP_ for each composition, see statistical detail in Supplementary Fig. 3). The local structures presented here can address the nanoscale structural features of the PNRs in BNT-based relaxor system, including the shape, size, and existing form.

Note here that the underlying nanoscale polar structure of BNT-RD12 contrasts with the conventional proposed “PNRs” model in lead-based relaxor ferroelectric systems assuming PNRs within nonpolar matrix^18^, but fits more to the model of local structural heterogeneity assuming the PNRs are embedded in the ferroelectric matrix^19^. Although the nanoscale polar structures of BNT-RD26 and BNT-RD38 (small-size polar structures connected by many low-angle walls) are consistent with the model of polar slush state^6,20^, a high density of high-angle domain walls can also be observed from the PFM images (Fig. 1a). This observation is also consistent with the diffuse scattering phenomenon in the BNT-based system, where the scattering pattern was observed to undergo a conformational change with increasing doping concentration^13,21^.

Another long-term controversy for the BNT-based relaxor system is the existence of local antiferroelectric (AFE) ordering^13,22^. As established in Pb-based relaxor systems, AFE ordering was considered to play an essential role in the relaxor physics and the superior dielectric/piezoelectric properties^2^, however, this critical configuration has not been experimentally corroborated in BNT-based relaxor system^13^. To validate the existence of local AFE ordering and gain insight into the dielectric relaxation behavior in the BNT-based system, we then analyzed the relative offset of cations from their adjacent anion centroid (Fig. 2c). The generally acknowledged AFE theory, proposed by Kittel, defines the AFE configuration as neighboring lines of cation–anion dipoles pointing in antiparallel directions^23^. After comparing the polar plots of the A–O/B–O displacement data (Fig. 2d) with those in ideal AFE and FE conditions (Supplementary Fig. 4), the absence of local AFE ordering is demonstrated in the BNT-based system, which is in good agreement with previous diffraction and scattering studies, indicating a lack of structural evidence for AFE in the BNT-based system^13^. The [110]_p_ projected structural information (Fig. 2b, d) is also verified by the [100]_p_ projected data (see Supplementary Fig. 5). Despite the absence of AFE ordering, it is worth noting that A-site cations exhibit the multidirectional ferro-distortive displacement behavior (see Supplementary Fig. 6), which is enhanced with increasing doping concentration, being discussed below.

After excluding the local AFE ordering, the short-range-correlated ferroelectric ordering (coupled polar regions) is further studied to explore the underlying physics and its contribution to relaxor ferroelectric properties. Characteristic parameters reflecting the relaxor degree (Fig. 2e) and the deviation degree of local polarizations (Fig. 2f) are compared. Three observations can be summarized with increasing doping concentration: (1) the dielectric constant increases with increased relaxation degree, while the *T*_d_ exhibits the opposite trend; (2) the polarization magnitude contributed by the relative B–O displacement gradually decreases (see decreased *δ*_B-O_/*δ*_A-O_); (3) the deviated polarization regions grow into larger regions (increased *R*_DP_) with smaller deviation angles (decreased *θ*_DP_). These features demonstrate a close relationship between the relaxor phenomenon and local polarization deviation, as shown in Fig. 2b. Although this atomic-scale structural configuration describes the composition-induced polarization evolution in the BNT-based system, the underlying physics that drive the formation of these deviated polarizations remains to be explored.

### Correlating the atomic-scale orderings to polarization deviation

The recent neutron scattering and STEM experiments demonstrate that three local orderings, including oxygen octahedral tilt (OT), distortion (OD), and chemical ordering (CO) may hold the key to engineer the local structures and thus the material properties in perovskite relaxor ferroelectrics^2,7^. In this study, based on data involving atomic position and intensity, we reveal these atomic-scale orderings in real space and understand their contributions to the local polarization.

**Fig. 3:**
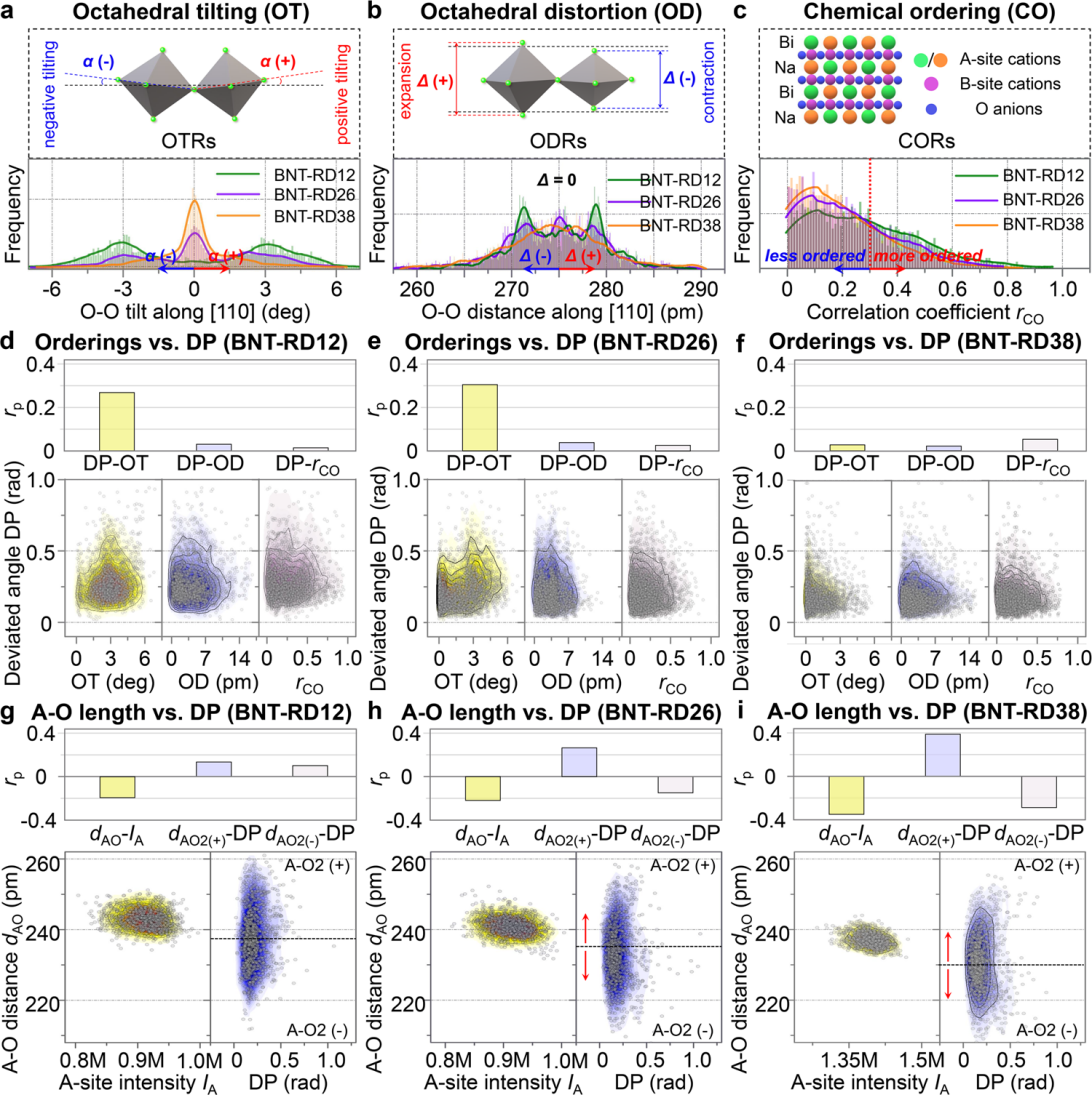
Correlation analysis between local orderings and the deviated polarization. **a**–**c** Schematic illustration (top), and statistical distribution (bottom) of local orderings in the BNT-based system, including **a** oxygen octahedral tilting (OT), **b** oxygen octahedral distortion (OD), **c** chemical ordering (CO). **d**–**f** Statistical point-to-point correlation analysis revealing the relationship between three local orderings and the deviated polarization. **g**–**i** Statistical point-to-point correlation analysis indicating the effects of A–O interactions on the deviated polarization changes with the doping concentration. *r*_p_ is the Pearson’s correlation coefficient, see detailed correlation data in Supplementary Table 1.

The position data of cation and oxygen atomic columns detected from STEM-ABF images provide feasibility to evaluate the ordering degree of OT (negative/positive) and OD (expansion/contraction)^7^, as schematically shown on the top of Fig. 3a, b (see details in Supplementary Note 3.2 and Supplementary Fig. 7a, b). Due to the sensitivity to the chemical distribution of cations, ADF STEM data were used to reveal the local Bi^3+^/Na^+^ distribution and evaluate CO of the local regions (schematically shown on the top of Fig. 3c, see details in Supplementary Note 3.3 and Supplementary Figs. 7c and 8)^7^. The distribution data of the three orderings are plotted as the frequency curves for a direct comparison (bottom of Fig. 3a–c), based on the statistical analysis (Supplementary Figs. 7 and 8) to quantify the relative fraction of these local orderings in different compositions.

The OT, OD, and CO decrease with increasing doping concentration (Fig. 3a–c). OT discussed here is the anti-phase octahedral tilting^7,24^. The decreased anti-phase OT suggests the reduced ratio of the local rhombohedral symmetry *R*3*c*, which follows the same trend as reported in previous X-ray, electron, and neutron diffraction and scattering results^13,14,21^. The anti-phase OT has been proven to be closely linked to the *T*_d_ based on neutron scattering results, suggesting a close relationship between this local feature and the stabilized ferroelectric polarization, where the decreased anti-phase OT accounting for the decreased *T*_d_. Interestingly, there is a cooperative relationship between the ferroelectricity and the anti-phase OT, which may originate from the internal polar field-enhanced higher-order coupling between them^25–29^. Meanwhile, the decreased CO indicates that the correlated Bi^3+^/Na^+^ arrangement is suppressed by chemical doping, which explains the progressively evolving diffused scattering profile^13,21^ and supports the result from the Monte Carlo simulation assuming the existence of a short-range ordered/disordered arrangement of Bi^3+^/Na^+^^30^.

Next, we link these local heterogeneities to the polarization deviation. By finding the local maxima of OT, OD, and CO, positions of these local heterogeneities are located (Supplementary Fig. 9)^7^, which are found to have the closer distance to low-angle walls (red regions) than randomly generated points. The point-to-point correlation analysis (Supplementary Note 3.4) is then used to reveal the dominant factors that drive the polarization deviation, as shown in Fig. 3d–f. Statistically, for BNT-RD12 and BNT-RD26, the OT is positively correlated with the deviated polarization (Fig. 3d, e), while the other two heterogeneities do not exhibit an obvious correlation with the deviated angle. Of particular significance is that the OT is proportional to the magnitude of B–O displacement (Supplementary Fig. 10). In other words, the larger the polarization induced by the relative B–O displacement, the larger the anti-phase OT. For BNT-RD38, on the other hand, the close correlation between OT and deviated polarization is disappeared (Fig. 3f). The magnitude of B–O displacement in BNT-RD38 is reduced (Supplementary Figs. 11–13), causing the loss of its correlation to the OT (Supplementary Fig. 10). Generally, the stable ferroelectric ordering (higher *T*_d_) is accompanied with the anti-phase OT. Thus the composition-induced decrease or disappearance of anti-phase OT leads to an unstable ferroelectric ordering (lower *T*_d_)^13,14,31,32^, which is attributed to the composition-induced difference in relative B–O displacements.

Interestingly, the red regions with deviated polarization in BNT-RD38 show a larger size (Fig. 2b), regardless that the OT contribution to the deviated polarization disappears, indicating that the driving force for polarization deviation is changed. Considering that the polarization is based on the relative cation–anion displacement, the relative A–O displacement is expected to modulate the polarization when the contribution from relative B–O displacement decreases. Correlation analysis on the deviation degree of polarization and A–O2 bond length (see details of choosing A–O2 in Supplementary Note 3.5 and Supplementary Figs. 11–13), shown in Fig. 3g–i, is used to evaluate the contribution of relative A–O displacement to the deviated polarization. As the doping concentration increases, it is interesting to note that the larger difference in A–O bond lengths (see details of A–O2 and A–O3 in Supplementary Figs. 11–13) accounts for the larger deviation degree of polarization in BNT-RD26 and BNT-RD38, which originates from the enhanced multidirectional displacement of A-site cations as discussed above.

### Underlying physics connecting local structures and material performance

We now use first-principles calculation and phase-field simulation to understand the underlying mechanisms and connect these local structures to material properties. For 2 × 2 × 2 perovskite unit cells of BNT, the total energy of different phases (cubic, rhombohedral, tetragonal) as a function of perovskite cell volume (see details in Supplementary Information Section 2.1 and Supplementary Figs. 14 and 15) is calculated. With the increase in cell volume, BNT tends to evolve from the rhombohedral phase into a tetragonal phase. Taking the cubic phase as the initial state, we calculate the cell energy against the displacement of Bi/Na/Ti atoms towards [001] and [111] directions. The Bi-O is found to shift preferentially along [001] and [111] directions while Ti-O along [111] direction, resulting in a multi-site probability density distribution for the displacement of the cations and anions. It can be concluded that through judicious chemical modification to increase the cell volume, the decisive effect of B–O relative displacement on the polarization direction is weakened, while the multidirectional relative A–O displacement has a greater contribution to the polarization direction.

**Fig. 4:**
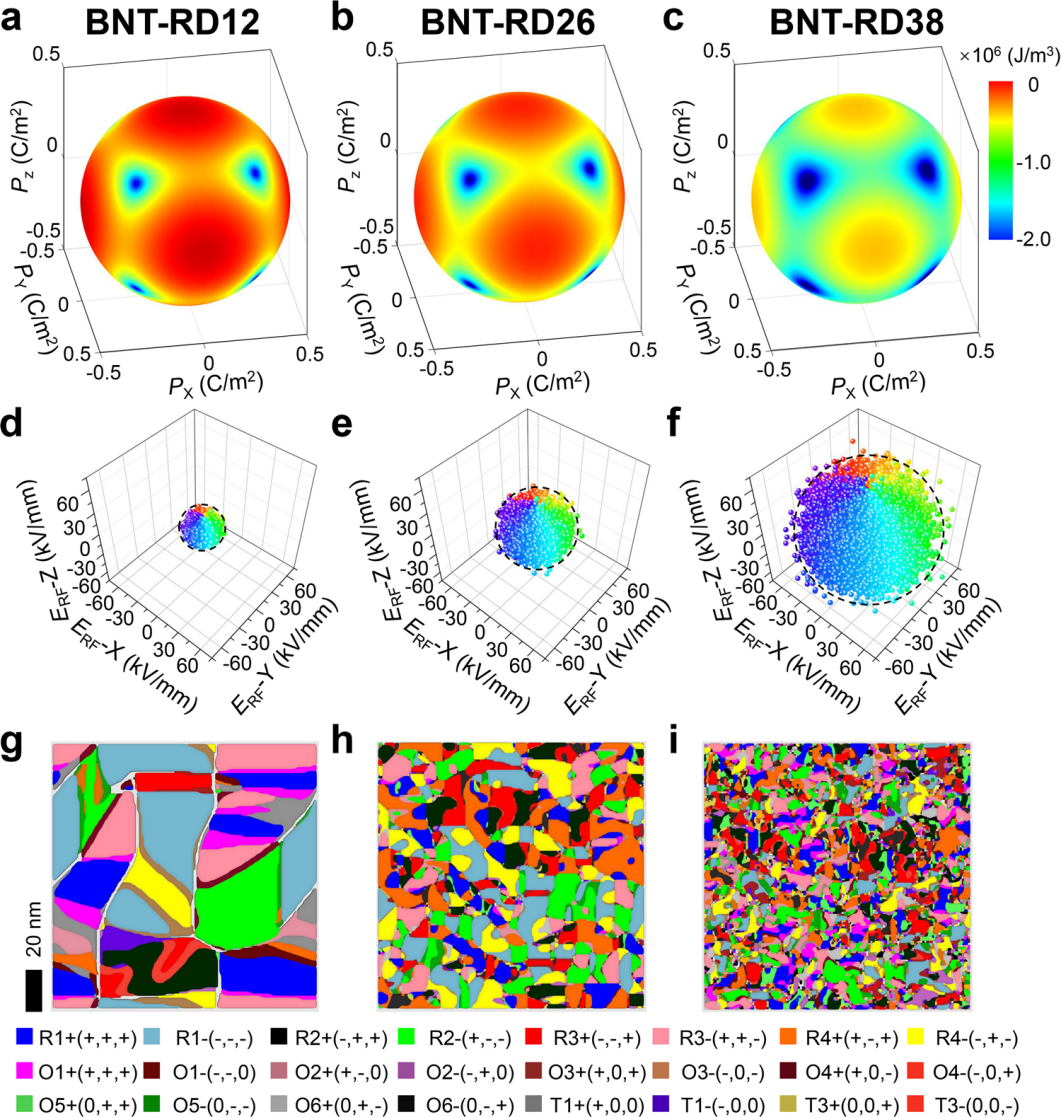
Evolution of polar structures simulated by the phase-field model. **a**–**c** Landau free-energy profiles, **d**–**f** random field distribution, and **g**–**i** simulated domain structures for (**a**, **d**, **g**) BNT-RD12, (**b**, **e**, **h**) BNT-RD26 and (**c**, **f**, **i**) BNT-RD38 at 25 °C. The axes in (**a**–**c**) represent the polarizations along [100], [010], [001] directions, and the value of energy density is described by the color and shown in the color bar. The axes in (**d**–**f**) represent the random field vectors (***E***_RF_) along [100], [010], [001] directions, and the color contrast describes the [001]_p_ projected vector angle of ***E***_RF_. R, O, T in the bottom of (**g**–**i**) represent rhombohedral, orthorhombic, and tetragonal symmetries.

The first-principles calculations help to understand the above STEM results at the atomic scale, while linking these local details to polar structures and material properties requires a mesoscale simulation method^33^. Based on the phase-field simulation, it can be found that the competition and coordination between the anisotropy field and random field dominate the evolution of polar structures and electromechanical properties. The decreased anisotropy field is introduced to describe the gradual weakening of B–O displacement and the enhancement effect of multidirectional A–O displacement on the polarization, while the increased spatially distributed defect-induced built-in random field is introduced to describe the increase in high-angle domain wall density, as shown in Fig. 4a–f (see details in Supplementary Information Section 2.2). The polar domain structures are obtained (Fig. 4g–i) by solving the time-dependent Ginzburg–Landau equation, consistent with the results experimentally imaged by PFM (Fig. 1a). The decreased anisotropy field leads to the flattened free-energy profile, which explains the enhanced dielectric constant, increased relaxor degree (Fig. 2e), and increased ratio of deviated polarizations with low-angle walls (Fig. 2b, f). On the other hand, the increased random field provides a larger driving force to restore the *E*-field-induced large-size domain to the multi-domain state (Supplementary Fig. 16), leading to a recoverable *E*-field-induced relaxor ferroelectric state and the so-called depolarization phenomenon (Supplementary Figs. 17 and 18). Notably, by increasing the random field or reducing the anisotropic field, *d*_33_^*^ increases first, and then decreases (Supplementary Fig. 19). Therefore, the optimized *d*_33_^*^ in the BNT-based system is attributed to the competitive and synergetic balance between anisotropy field and random field.

**Fig. 5:**
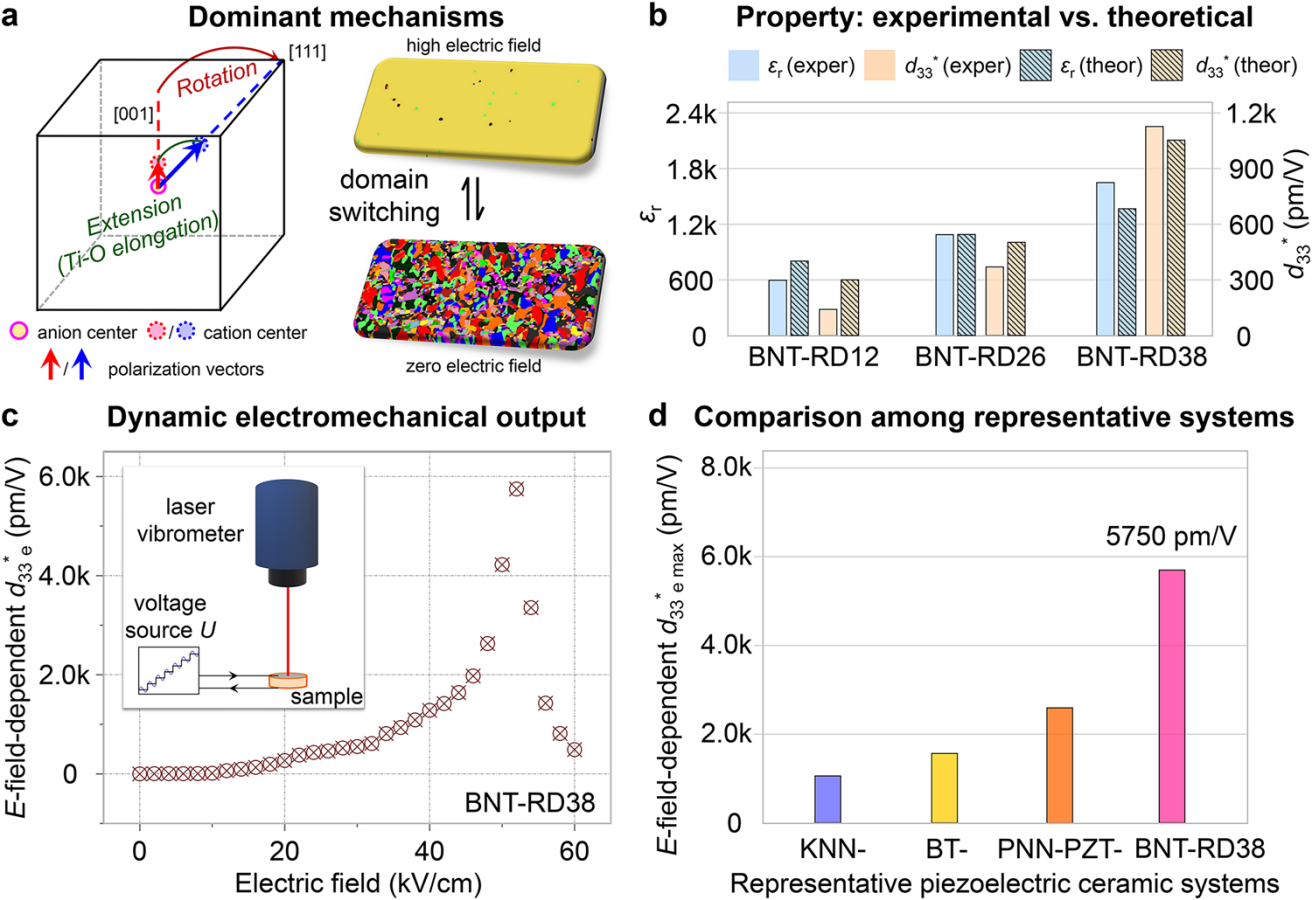
Dominant mechanisms for the large dynamic electromechanical response and the potential for applications. **a** Schematic illustration of the dominant mechanisms (polarization rotation, extension, and domain switching) for the large dynamic electromechanical response in the BNT-based system. **b** Comparison of representative material properties (dielectric constant *ε*_r_ and dynamic piezoelectricity *d*_33_^*^) from experimental and theoretical perspectives. **c** Dynamic electromechanical output reflected by the *E*-field-dependent effective piezoelectric strain coefficient *d*_33_^*^_e_. The inset shows the schematic illustration of how to measure the *E*-field-dependent *d*_33_^*^_e_, see details in Supplementary Information 1.2. **d** Comparison of the maximum of *E*-field-dependent *d*_33_^*^_e_ in representative electromechanical ceramic systems. Details can be seen in Supplementary Fig. 22.

Based on the in situ *E*-field-dependent synchrotron XRD and selected area electron diffraction patterns (Supplementary Fig. 20), the *E*-field-induced polarization rotation from tetragonal *P*4*bm* to rhombohedral *R*3*c* is demonstrated. Our point-to-point correlation analysis has revealed that the rhombohedral *R*3*c*, corresponding to the anti-phase OT, is highly correlated with the magnitude of B–O displacement. In this scenario, the increased *R*3*c* component reflects the elongated B–O displacement, indicating a polarization extension occurs under the applied *E*-field. Meanwhile, the effect of B–O displacement on the total polarization is gradually weakened with increasing the doping concentration. While the impact of multidirectional A–O displacement is enhanced, which reduces the anisotropy field between different symmetries, being conducive to the facilitated polarization rotation and extension. Combined with the voltage-induced evolution of domain structures and related strain behaviors, for the system reaching a critical equilibrium between the anisotropy field and the random field, a significantly increased strain occurs during the domain switching process. Therefore, we demonstrate that three dominated factors are responsible for the enhanced dynamic electromechanical response, including polarization rotation, polarization extension, and domain switching (Fig. 5a). This can be confirmed by the experimental measurement and phase-field simulated properties (Fig. 5b and Supplementary Fig. 21). Of particular interest is that at a specific *E*-field level, most of the polarizations will rotate and/or extend together with the reversible domain switching process, leading to an ultrahigh electromechanical response (Fig. 5c, *d*_33_^*^_e_ = 5750 pm/V, see details in Supplementary Methods 1.2). Figure 5d compares the maximum *E*-field-dependent *d*_33_^*^_e_ in representative high-performance electromechanical ceramic systems (see details in Supplementary Fig. 22), where BNT-RD38 shows a great potential for electromechanical applications.

## Discussion

We now discuss how to achieve the optimized dynamic electromechanical response. For the BNT-based relaxor system, a common sense to enhance its dynamic electromechanical response is shifting the *T*_d_ to ambient temperature by chemical modifications. However, despite the same *T*_d_ shift, different kinds of doping lead to significantly different dynamic electromechanical responses (see the detailed comparison in Supplementary Fig. 23). Three general observations are summarized in the following: (1) the BNT-based material with higher piezoelectric charge coefficient *d*_33_ is easier to achieve a larger piezoelectric strain coefficient *d*_33_^*^ by doping; (2) heterovalent cation doping is more effective in decreasing *T*_d_ and enhancing *d*_33_^*^ than isovalent cation doping; (3) in contrast to A-site doping, B-site doping requires a much lower doping concentration to shift *T*_d_ to the ambient temperature. All these observations can be explained based on the local structural and chemical features of BNT-based relaxor system.

For perovskite ferroelectric solid solutions, the enhanced small-signal piezoelectricity is attributed to the improved dielectric permittivity^34^, originating from the decreased anisotropy field and thus the flattened free-energy profile. We demonstrate that the deviated polarizations with low-angle walls play a vital role in regulating the anisotropy field. By reducing the anti-phase OT (corresponding to the decreased rhombohedral *R*3*c* symmetry) and enhancing the impact of multidirectional A–O displacement, the magnitude of polarization is reduced, and more polarizations with low-angle walls deviate from their surroundings with the decreased *θ*_DP_, leading to an effectively reduced anisotropy field and increased dielectric permittivity. This can be achieved by introducing a tetragonal component whose A-site cations have the potential to shift in multi-directions. Our first-principles calculation (Supplementary Fig. 15) shows that for Bi_0.5_K_0.5_TiO_3_ (BKT), there is a small energy gap between [111] and [001] displacement directions of Bi^3+^, while the overall polarization tends to shift towards the tetragonal [001] direction. Therefore, BNT-BKT composition is an ideal matrix composition to further decrease the anisotropy field and flatten the energy gap between different symmetries. By applying an *E*-field, the weakened anisotropy field facilitates the polarization rotation from *P*4*bm* to *R*3*c* (accompanied with the polarization extension). On the other hand, as observed from the deviated polarizations with high-angle walls, the doping-introduced heterovalent defects effectively regulate the random field, providing the restoring force from *E*-field-induced large-size ferroelectric domains to small-size multi-domains. The polarization analysis indicates that the relative B–O displacement dominates the total polarization, demonstrating that the defects introduced through heterovalent B-site doping are more effective in regulating the random field and their surrounding polarizations. The heterovalent doping on A-site, although not as efficient as that on B-site, has a similar effect in regulating the random field, due to the Bi/Na-Ti interactions mediated by oxygen displacement. Notably, reducing the anisotropy field and/or increasing the random field will both lead to the depolarization phenomenon in BNT-based relaxor system, suggesting that *T*_d_ can be shifted to the ambient temperature in different ways. As discussed above, an optimized dynamic large-signal electromechanical response can only be achieved when the anisotropy field and random field reach a critical equilibrium, where the competitive and synergetic effects between the anisotropy and random fields should be considered. This can be realized by the chemical modifications, which are expected to greatly impact the local structural features (see the framework of composition–structure-performance correlation in Supplementary Fig. 24). In addition to chemical modifications, it is worth noting that modifying the material synthesis conditions (such as quenching^35–37^, annealing^38^, sintering temperature^39^, sintering dwell time^39^, and sintering atmosphere^15^, to name a few) is also the common approach to manipulate the local structural features, providing more freedom to optimize the dynamic electromechanical response.

In summary, with both experimental and theoretical analyses, we successfully establish the composition–structure-property correlation in the relaxor ferroelectric BNT-based system. The large electromechanical property achieved in this work, originating from the thermodynamic behavior of deviated polarizations, shows the potential for dynamic electromechanical applications. From the phenomenological perspective, the critical ferroelectric state consists of different deviated polarizations with high-angle walls and low-angle walls, which is the result of a competitive and synergetic equilibrium between the anisotropy field and random field. We demonstrate that doping-induced local chemical/structural evolution provides the main driving force for polarization deviation. Therefore, to design materials with larger dynamic electromechanical response, balancing the local chemical/structural orders that can drive the polarization deviation is the key. This can be achieved by judicious selection of the matrix oxides, introduction of suitable dopants, and modification of synthesis conditions.

## Methods

See details of the methods part in the Supplementary Information files.

## Supplementary information

Supplementary Information

## Data Availability

The data corresponding to this study are available from the first author and corresponding authors upon request.

## Code availability

MATLAB scripts are available from the first author and corresponding authors upon request.
