# Author Correction: Deciphering the atomic-scale structural origin for large dynamic electromechanical response in lead-free Bi_0.5_Na_0.5_TiO_3_-based relaxor ferroelectrics

**DOI:** 10.1038/s41467-022-34663-1

**Published:** 2022-11-09

**Authors:** Jie Yin, Xiaoming Shi, Hong Tao, Zhi Tan, Xiang Lv, Xiangdong Ding, Jun Sun, Yang Zhang, Xingmin Zhang, Kui Yao, Jianguo Zhu, Houbing Huang, Haijun Wu, Shujun Zhang, Jiagang Wu

**Affiliations:** 1 Department of Materials Science, Sichuan University, Chengdu, China; 2 Institute of Materials Research and Engineering, Agency for Science, Technology and Research (A*STAR), Singapore, Singapore; 3 State Key Laboratory for Mechanical Behavior of Materials, Xi’an Jiaotong University, Xi’an, China; 4 Advanced Research Institute of Multidisciplinary Science, Beijing Institute of Technology, Beijing, China; 5 Physics Department, Southwest Minzu University, Chengdu, China; 6 Instrumental Analysis Center of Xi’an Jiaotong University, Xi’an Jiaotong University, 710049 Xi’an, China; 7 Shanghai Synchrotron Radiation Facility, Shanghai Institute of Applied Physics, Chinese Academy of Sciences, Pudong New Area, Shanghai, China; 8 Institute for Superconducting and Electronic Materials, Australian Institute of Innovative Materials, University of Wollongong, Wollongong, NSW Australia

**Keywords:** Electronic properties and materials, Ferroelectrics and multiferroics

Correction to: *Nature Communications* 10.1038/s41467-022-34062-6, published online 25 October 2022

In this article the affiliation ‘Institute of Materials Research and Engineering, Agency for Science, Technology and Research (A*STAR), Singapore, Singapore’ for Jie Yin was missing. The original article has been corrected.

