# Author Correction: Deciphering the atomic-scale structural origin for large dynamic electromechanical response in lead-free Bi_0.5_Na_0.5_TiO_3_-based relaxor ferroelectrics

**DOI:** 10.1038/s41467-023-37006-w

**Published:** 2023-03-17

**Authors:** Jie Yin, Xiaoming Shi, Hong Tao, Zhi Tan, Xiang Lv, Xiangdong Ding, Jun Sun, Yang Zhang, Xingmin Zhang, Kui Yao, Jianguo Zhu, Houbing Huang, Haijun Wu, Shujun Zhang, Jiagang Wu

**Affiliations:** 1 Department of Materials Science, Sichuan University, Chengdu, China; 2 State Key Laboratory for Mechanical Behavior of Materials, Xi’an Jiaotong University, Xi’an, China; 3 Advanced Research Institute of Multidisciplinary Science, Beijing Institute of Technology, Beijing, China; 4 Physics Department, Southwest Minzu University, Chengdu, China; 5 Instrumental Analysis Center of Xi’an Jiaotong University, Xi’an Jiaotong University, 710049 Xi’an, China; 6 Shanghai Synchrotron Radiation Facility, Shanghai Institute of Applied Physics, Chinese Academy of Sciences, Pudong New Area, Shanghai, China; 7 Institute of Materials Research and Engineering, Agency for Science, Technology and Research (A*STAR), Singapore, 138634 Singapore; 8 Institute for Superconducting and Electronic Materials, Australian Institute of Innovative Materials, University of Wollongong, Wollongong, NSW Australia

**Keywords:** Electronic properties and materials, Ferroelectrics and multiferroics

Correction to: *Nature Communications* 10.1038/s41467-022-34062-6, published online 25 October 2022

In this article the affiliation ‘Institute of Materials Research and Engineering, Agency for Science, Technology and Research (A*STAR), Singapore, Singapore.’ for Jie Yin should be removed. The affiliation ‘Institute of Materials Research and Engineering, Agency for Science, Technology and Research (A*STAR), Singapore, Singapore’ for Kui Yao should read “Institute of Materials Research and Engineering, Agency for Science, Technology and Research (A*STAR), Singapore 138634, Singapore”. The original article has been corrected.

