# Supplementary material for: Deciphering the atomic-scale structural origin for large dynamic electromechanical response in lead-free Bi0.5Na0.5TiO3-based relaxor ferroelectrics
Source: Nat Commun. 2022 Oct 25;13:6333. doi: 10.1038/s41467-022-34062-6 (PMC9596697; doi:10.1038/s41467-022-34062-6)
Supplement: Supplementary file 1 — Supplementary Information [file 41467_2022_34062_MOESM1_ESM.pdf]

Supplementary Information for

Deciphering the atomic-scale structural origin for large dynamic electromechanical response in lead-free $Bi_{0.5}Na_{0.5}TiO_3$-based relaxor ferroelectrics

Jie Yin[1,†], Xiaoming Shi[2,3,†], Hong Tao[1,4], Zhi Tan[1], Xiang Lv[1], Xiangdong Ding[2], Jun Sun[2], Yang Zhang[5], Xingmin Zhang[6], Kui Yao[7], Jianguo Zhu[1], Houbing Huang[3], Haijun Wu[2,✉], Shujun Zhang[8,✉], Jiagang Wu[1,✉]

[1]Department of Materials Science, Sichuan University, Chengdu, China. [2]State Key Laboratory for Mechanical Behavior of Materials, Xi'an Jiaotong University, Xi'an, China. [3]Advanced Research Institute of Multidisciplinary Science, Beijing Institute of Technology, Beijing, China. [4]Physics Department, Southwest Minzu University, Chengdu, China. [5]Instrumental Analysis Center of Xi'an Jiaotong University, Xi'an Jiaotong University, Xi'an 710049, China. [6]Shanghai Synchrotron Radiation Facility, Shanghai Institute of Applied Physics, Chinese Academy of Sciences, Pudong New Area, Shanghai, China. [7]Institute of Materials Research and Engineering, Agency for Science, Technology and Research (A*STAR), Singapore. [8]Institute for Superconducting and Electronic Materials, Australian Institute of Innovative Materials, University of Wollongong, Wollongong, New South Wales, Australia. [†]These authors contributed equally: Jie Yin, Xiaoming Shi. [✉]e-mail: wuhaijunnavy@xjtu.edu.cn; msewujg@scu.edu.cn; shujun@uow.edu.au

1. Methods

1.1 Sample Fabrication

BNT-based relaxor ferroelectric ceramic samples were prepared by solid-state reaction method with Bi_2O_3 (99.0%), Na_2CO_3 (99.8%), K_2CO_3 (99.0%), Li_2CO_3 (98.0%), TiO_2 (99.5%), Ta_2O_5 (99.999%) as raw materials to fabricate. The chemical formulas of $Bi_{0.50}Na_{0.50}TiO_3$, $Bi_{0.50}(Na_{0.72}K_{0.18}Li_{0.10})_{0.50}TiO_3$, and $Bi_{0.50}(Na_{0.72}K_{0.18}Li_{0.10})_{0.50}Ti_{0.985}Ta_{0.015}O_3$ are abbreviated as BNT-RD12, BNT-RD26 and BNT-RD38, respectively. After these raw materials were weighed according to the chemical formulas, they were ball milled for 24 h with both Y-stabilized ZrO_2 balls and ethyl alcohol as the media. The dried powders were then calcined at 900 °C for 6 h. 5 *wt* % polyvinyl alcohol (PVA) was used as the mixing binder to help the calcined powders be pressed into pellets (uniaxial pressure: 10 MPa, diameter: 10.0 mm, thickness: 1.0 mm). After the removal of PVA (furnace heating temperature: 550 °C), cold isostatic pressing was loaded on the samples (isostatic pressure: 300 MPa). The pellets were buried in their corresponding calcined powders and sintered at 1130 °C for 3 h (air atmosphere sintering, cooling rate: 10 °C/min). The relative density of the ceramics was measured by the Archimedes method, and all ceramics possess high relative densities (>95%). The silver paste was pasted on both sides of the sintered and polished pellets (500 μm) to characterize the electrical properties.

1.2 Electrical Property Measurement

Poling treatment was applied by using a D.C. (direct current) electric field (*E*-field: 6 kV/mm) in a silicon oil bath. Berlin-court method (piezo-meter, ZJ-3A, China) was used to measure the quasi-static d_{33} values. The high-resolution frequency- and temperature-dependent dielectric signals (ε_r and tan δ, 100 Hz–500 kHz, -160 °C-300 °C) were characterized by the commercial precisely controlled dielectric spectrometer instrument (Novocontrol Concept 80, Novocontrol, Germany). *In-situ E*-field-dependent bipolar polarization, electro-strain, and unipolar stain (bipolar *P-E*, *S-E*, unipolar *S-E*) curves were measured by the ferroelectric tester (aixACCT TF Analyzer 2000, Germany) with the frequency of 1 Hz and the temperature of 25 °C. The *E*-field-dependent effective piezoelectric strain coefficient $d_{33}{}^*{}_e$ was determined at room temperature by measuring the displacement difference ($\triangle z = z_i - z_{i-1}$) between two stepped voltage ($\triangle U = U_i - U_{i-1}$) as $\triangle z / \triangle U$, using the laser vibrometer.

1.3 Structural Characterization

1.3.1 Piezo-force Microscopy (PFM)

Before the polishing treatment on the sintered samples, they were ground down to the thickness of 400 μm. Then, they were polished to have a flat surface using polycrystalline diamond paste with abrasive particles of 9 μm, 3 μm, 1 μm, and 0.3 μm (DP-Paste P by Struers A/S, Ballerup, Denmark). The domain morphology, local poling experiment, and local switching spectroscopy were obtained on the atomic force microscope (AFM, MFP-3D, Asylum Research, USA) with PFM functions. The driving voltage (1.5 V) was applied on the Pt- and Ir-coated cantilever (NanoWorld EFM, force constant: 2.8 N/m, resonance frequency: 75 kHz) to obtain the domain morphology.

1.3.2 In-situ electric field-dependent synchrotron radiation X-ray diffraction (in-situ *E*-field-dependent synchrotron XRD)

In-situ *E*-field-dependent synchrotron XRD experiments on BNT-based samples were performed at the Shanghai Synchrotron Radiation Facility using a beam line 14B1 (λ = 1.2521 Å). Samples were ground down to the thickness of 500 μm. To measure the *E*-field-dependent average structures, a platinum electrode layer was coated on the two sides of BNT-based samples. Before the test, the samples were dried at 150 °C for 6 h. By using the high-voltage generator with the DC working mode, the *E*-field was applied on the sample.

1.3.3 (Scanning) Transmission Electron Microscopy (TEM/STEM)

Samples used for (S)TEM investigation were prepared by slicing, polishing, and then ion milling with liquid N_2 cooling (Fischione M1051 TEM Mill). Aberration-corrected STEM characterization was conducted with a JEOL ARM200F equipped with a cold field emission gun and ASCOR probe corrector.

Capturing the data of atomic position and intensity of cations and anions

Supplementary Fig. 1a shows the projection of cubic ABO_3 (*Pm-3m*) unit cell along the [110] zone axis, which is the reference structure in this work. As shown in Supplementary Fig. 1b, due to its sensitivity to chemical distribution, annular dark-field scanning transmission electron microscopy (ADF STEM) is used to capture the position and intensity of cations. The light element, oxygen, is captured by the annular bright-field (ABF) and integrated differential phase contrast (iDPC) STEM. By simultaneously capturing ADF and ABF-iDPC[1,2] STEM images, the position and intensity of cations and anions in their corresponding sublattices can be technically detected, which enables the atomic-scale point-to-point correlation analysis. The local rhombohedral $R3c$ symmetry can be characterized by the anti-phase oxygen octahedra tilt patterns, as described by the rotation angle θ along the pseudo-cubic $<110>_{pc}$ axis shown in Supplementary Fig. 1c[3]. By choosing the [110] direction as the zone axis, the correlation between the $R3c$ symmetry, the local octahedral structure, local polarization, and material properties can be established in $Bi_{0.5}Na_{0.5}TiO_3$-based relaxor ferroelectrics.

Quantifying the projected polarization

For the $[110]_p$ projected STEM images, the overlapping effect of A-site cations and oxygen is required to be discussed[2]. According to Kumar *et al.*'s work[2], the captured positions for A-site cations are dominated by the displacement of A-site cations, validating the feasibility of analyzing the details of A-site cations by using the position and intensity data detected from ADF STEM. Then, the local polarization vectors are evaluated from the displacement between the B-site cations and the octahedra center, $\delta_{B\text{-}O}$, based on a linear relationship $P_s = k\delta_{B\text{-}O}$ for displacive ferroelectrics, where k is a material-dependent proportionality constant[4,5]. For each B-site cation, the centroid of the two nearest oxygen atom columns is used as the center of the oxygen octahedra, as shown in Supplementary Fig. 1d. This is a generally accepted method to evaluate the polarization in displacive ferroelectrics, like $PbTiO_3$[6], Pb(Zr, Ti)O_3[7], $BiFeO_3$[8], $SrTiO_3$[9], $BaTiO_3$[10], (K, Na)NbO_3[11], (La, Sr)MnO_3[12], etc. The atomic positions of the atomically resolved STEM images are located accurately by fitting them as 2D Gaussian peaks.

The visualization of the two-dimensional polar displacement vectors with the information of magnitude and direction was carried out using Matlab.

2. Theoretical Calculations and Simulations

2.1 First-principles calculation

In this work, the first-principles calculation is carried out using Vienna Ab initio Simulation Package (VASP), based on the density functional theory (DFT)[13-15]. The exchange-correlation functional is treated within the generalized gradient approximation (GGA) parameterized by Perdew-Burke-Ernzerhof revised for solids (PBEsol)[16]. The electron-ion potential is described by employing the projector augmented-wave (PAW) potential[17].

A 2×2×2 supercell with ABO_3 structure containing 40 atoms is built, to describe the *A*-site atomic orders of $Bi_{0.5}Na_{0.5}TiO_3$ and $Bi_{0.5}K_{0.5}TiO_3$, where the states of 5d6s6p for Bi, 2p3s for Na, 3s3p4s for K, 3s3p3d4s for Ti, and 2s2p for O are treated as valence electrons for calculations, and the random A-site atomic configuration is generated using the special quasi-random structure (SQS) approach[18]. The first Brillouin zone in the present work is sampled by adopting a 7×7×7 Monkhorst-Pack *k*-mesh. The wave function is represented as a plane wave expansion that is truncated at cut-off energy of 500 eV. The Kohn-Sham orbitals are updated in the self-consistency cycle until an energy convergence of 10^{-5} eV is obtained. All the geometry optimizations were completed when the residual force of each atom was less than 0.01 eV/Å. To investigate the relationship between total energy and atomic displacement, a linear interpolation is adopted in a fixed supercell. The force-constant of Ti along a certain direction is obtained via calculating the second derivative of energy to the atomic displacement according to:

$$K = \Omega_0 \frac{\partial^2 E}{\partial u^2} \tag{1}$$

where the Ω_0 is the volume, and u is the atomic displacement.

2.2 Phase-field simulations

Phase-field simulations of $Bi_{0.5}Na_{0.5}TiO_3$-based solid solutions are used to investigate the composition-dependent evolution of domain structure, material performance as well as intrinsic mechanisms. In the present work, the domain structures are described by the spatial distribution of spontaneous polarization $\boldsymbol{P}$ (P_1, P_2, P_3). The temporal evolution of the polarization is described by the time-dependent Ginzburg-Landau (TDGL) equation and the displacement field $\boldsymbol{u}$ can be described as the stress equilibrium equation[19-21],

$$\begin{aligned} &\frac{\partial P_i}{\partial t} + L\frac{\delta F}{\delta P_j} = 0 \\ &\frac{\partial}{\partial x_j}\left(\sigma_{ij}(\mathbf{r},t)\right) = 0 \end{aligned} \tag{2}$$

Here, L is a kinetic coefficient related to domain wall mobility, F is the total free

energy of the system, $\frac{\delta F}{\delta P_j}$ is the thermodynamic driving force for polarization evolution, σ_{ij} is the stress tensor, and r and t are the spatial coordinate and time, respectively. The total free energy of a bulk system can be defined as follows,

$$F = F_{bulk}(\boldsymbol{P}) + F_{wall}(\boldsymbol{P}) + F_{elastic}(\boldsymbol{P}) + F_{elec}(\boldsymbol{P}, \boldsymbol{E}) \quad (3)$$

Here, F includes the bulk free energy $F_{bulk}(\boldsymbol{P})$, domain-wall energy $F_{wall}(\boldsymbol{P})$, elastic energy $F_{elastic}(\boldsymbol{P})$, and electrostatic energy $F_{elec}(\boldsymbol{P}, \boldsymbol{E})$, where E is the applied static electric field.

The bulk free-energy density is expressed for zero strain as a six-order polynomial expansion, that is

$f_{bulk} = \alpha_1(P_1^2 + P_2^2 + P_3^2) + \alpha_{11}(P_1^4 + P_2^4 + P_3^4) + \alpha_{12}(P_1^2P_2^2 + P_2^2P_3^2 + P_1^2P_3^2) + \alpha_{111}(P_1^6 + P_2^6 + P_3^6) + \alpha_{112}[P_1^4(P_2^2 + P_3^2) + P_2^4(P_1^2 + P_3^2) + P_3^4(P_1^2 + P_2^2)] + \alpha_{123}P_1^2P_2^2P_3^2$ (4)

where α_i, α_{ij}, α_{ijk} are the Landau parameters that can be obtained from the experiment. The elastic energy density can be written as $f_{elas} = \frac{1}{2}C_{ijkl}(\varepsilon_{ij}-\varepsilon_{ij}^0)(\varepsilon_{kl}-\varepsilon_{kl}^0)$, where C_{ijkl} is the elastic stiffness tensor, ε_{ij} is the total strain and ε_{ij}^0 is the spontaneous strain during the phase transformation. The spontaneous strain is related to the polarization by the electrostrictive coefficients $\varepsilon_{ij}^0 = Q_{ijkl}P_kP_l$, where Q_{ijkl} is the electrostrictive coefficient. The anisotropy factor $A = C_{44}/C_{11}$. The gradient energy density can be obtained by $f_{grad} = \frac{1}{2}G_{ijkl}\, P_{i,j}\, P_{k,l}$, where G_{ijkl} is the gradient coefficient.

The electrostatic energy can be expressed as $f_{elec} = \frac{1}{2}(\boldsymbol{E}\cdot\boldsymbol{P})$, where $\boldsymbol{E}$ is the total electric field, which can be described as, $E = E_{appl} + E_{dipole} + E_{RF}$, where E_{appl} is the applied electric field, E_{dipole} is the dipole-dipole interaction field, and E_{RF} is the local electric field caused by the random point defects. The dipole-dipole interaction electric field can be evaluated from the electrostatic potential φ as $\boldsymbol{E_{dipole}} = -\nabla\varphi$. In the model the space is assumed to be charge-free, therefore the equation: $\nabla\cdot(-\varepsilon_0\, \varepsilon_{br}\nabla\varphi+\boldsymbol{P}) = 0$ can be used to solve the electric field, where ε_0 and ε_{br} are vacuum and the background relative dielectric constants, respectively.

The parameters in details are provided as, $a_1 = 4.124\times10^5\,(T-T_0)$, $a_{11} = -5\times10^8\,(c-c_0)$, $a_{12} = 8.0\times10^8\,(c-c_0) + 2a_{11}$, $a_{111} = 1.294\times10^9\,(c-c_0)$, $a_{112} = 1.5\times10^9\,(c-c_0) + 3a_{111}$, $a_{123} = 7.5\times10^8\,(c-c_0) + 6a_{111}$, $Q_{11} = 0.1$, Q_{12}=-0.034, $Q_{44} = 0.029$, $s_{11} = 9.1\times10^{-12}$, s_{12}= -3.2×10^{-10}, $s_{44} = 8.2\times10^{-10}$. These parameters are all in SI units. And s is the compliance coefficients, T_0 is the curie temperature, c is the doping level and c_0 is the critical doping level. The above Landau coefficients can produce a transition from short-range ordered relaxor state to long-range-ordered R state with temperature lower than T_0, and the symmetry transition with increased doping level c ($c>c_0$).

With doping from BNT-RD12 to BNT-RD38, two effects are employed. The increased high-angle domain wall density is characterized by increasing the random

local defect concentration and random local electric field variance. Here, the random local defect will create nanoscale composition fluctuations, leading to Curie temperature variation and random local electric field. The Curie temperature variation is expressed as, $T_0 = T_{00} - bx$, where T_{00} is the Curie temperature without doping, b is a constant and x is the defect concentration. The strength of the random local electric field is assumed to decrease linearly with x.

The doping-induced weakened polarization originated from the relative B-O displacement component and the continuously connected polarization originated from the multi-directional displacement of A-site cations is characterized as decreasing the free energy barrier, which is governed by the doping level. The simulation scale is 256dx×256dz, and the grid scales dx and dz are 1 nm. The Fourier method was used for solving the equations.

3. Supplementary Notes

3.1 Supplementary Note 1: Evaluating the deviation degree of polarization

For each unit cell, its deviated polarization angle (θ_{DP}) is defined as, $\theta_{DP} = \sum_i |\theta_i - \theta_0| / 8$, where θ_0 is the polarization angle of the central unit cell and θ_i is the polarization angle of the unit cells surrounding the central unit cell[2], as schematically mentioned in Fig. 2a in the main text. A global median threshold is defined as: $M_\theta = \sum_i \theta_{DP,i}/i$, which is used to generate a binary mask ($\theta_{DP} > M_\theta$, = 1; $\theta_{DP} < M_\theta$, = 0) that can distinguish the polar regions with large and small deviation degrees[2].

3.2 Supplementary Note 2: Evaluating the tilting and distortion degree of Oxygen Octahedra

Oxygen octahedral tilting and distortion (expansion/contraction) degrees are evaluated based on the data from iDPC images, as schematically shown in Supplementary Fig. 1b and the main text (Fig. 3). To directly correlate the deviated polarizations with the degree of oxygen octahedral tilting and distortion, the tilted degree, and distortion degree are used to determine the octahedral tilting and distortion regions, as shown in Supplementary Fig. 7a, 7b. After the tilted angles and distortion values are calculated, they are plotted as a matrix form and statistically presented as the distribution form (Fig. 3 in the main text).

3.3 Supplementary Note 3: Analyzing the Chemical Ordering

The statistical correlation method is employed to evaluate the local chemical ordering of BNT-based system[2], as shown in Supplementary Fig. 8. First, the intensity data of A-site columns are normalized according to the average intensity of local 3×3 unit cells (Supplementary Fig. 8a)[2], and the normalized data are then subtracted from the local mean (Supplementary Fig. 8b)[2,22]. After that, a standard 3×3 correlation template (Supplementary Fig. 8c) is used to evaluate the chemical ordering of A-site cations, and the calculated data reflecting the regions with strong and weak chemical ordering are provided in Supplementary Fig. 8d. To better present the local ordering, data provided in Supplementary Fig. 8b are classified as “-1” and “+1” values, which

are based on whether they are negative or positive, as shown in Supplementary Fig. 8e. Then, correlation analysis is performed on these classified data to obtain the adjusted chemical ordering (Supplementary Fig. 8f). The chemical ordered regions are quantified by applying a threshold mask on the calculated data, as shown in Supplementary Fig. 8g-8i.

3.4 Supplementary Note 4: Spatial and Point-to-point Correlation Analysis

To evaluate the spatial relationship between chemical ordering, octahedral tilting degree, octahedral distortion degree, and deviated polarizations, the local ordering centers are obtained by finding the local maxima of these data, respectively. The local maxima are then used as the positions of these local ordering, and the filtered local polar structures are used as the background, as provided in Supplementary Fig. 9.

The maxima of these orderings seem to spatially correlate with the deviated polarizations, but the critical factor that dominates the deviation of the polarization cannot be obtained from this spatial image. Therefore, direct point-to-point correlation analysis (Pearson's method, using IBM SPSS Statistics 26, Python Extension Commands for SPSS Statistics) is then performed on these variables. Two variables are selected for each correlation analysis, including (1) Oxygen octahedral tilting angle *vs.* deviation angle of polarization, (2) oxygen octahedral distortion degree *vs.* deviation angle of polarization, (3) chemical ordering degree *vs.* deviation angle of polarization, and (4) bond length of A-O2 *vs.* deviation angle of polarization. Pearson's correlation coefficient r_p represents the correlation degree of two variables, ** is the symbol that indicates the significant correlation between two variables, sig is the significance factor.

3.5 Supplementary Note 5: Bond-length Analysis

A-O bond lengths are analyzed by calculating the distance between the four adjacent oxygen anions and A-site cations, while B-O bond lengths are analyzed by calculating the distance between the two adjacent oxygen anions and B-site cations. By statistically comparing the discrepancies between two bond lengths (A-O1 vs. A-O2, A-O3, A-O4 and A-O2 vs. A-O3, A-O4, B-O1 vs. B-O2), directions of the relative displacement between cations and anions can be presented more clearly, as provided in Supplementary Fig. 11-1. Two important features can be summarized from the bond-length analysis: (1) Multi-directional displacement can be observed for A-O; (2) The magnitude of B-O displacement is gradually reduced.

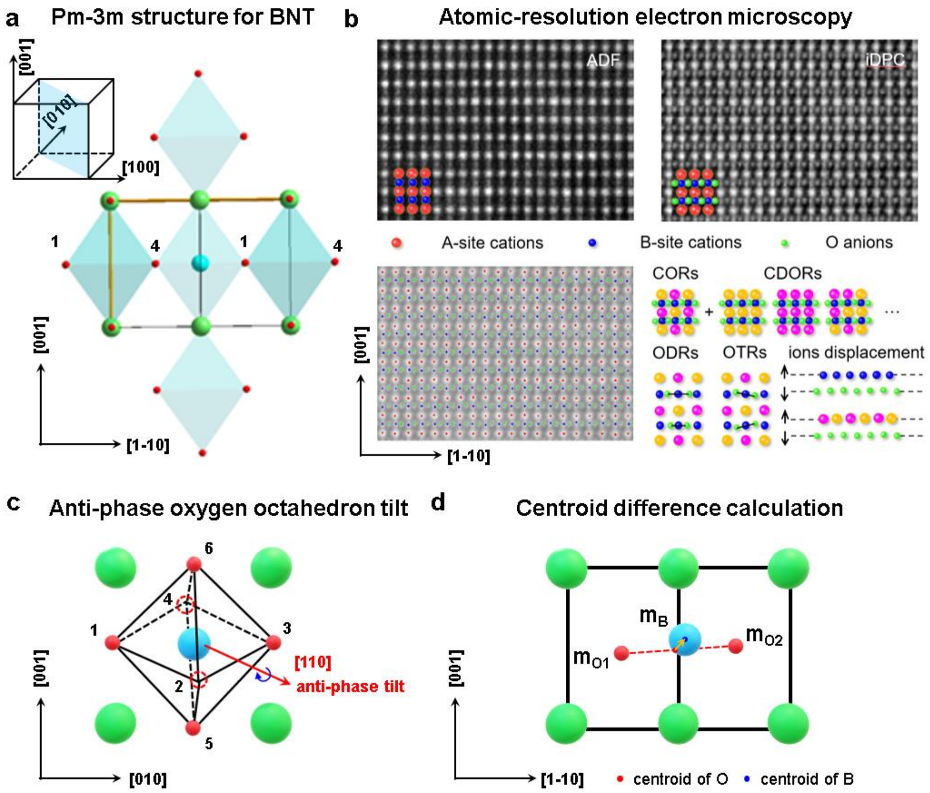

Supplementary Fig. 1 | Principles utilized to capture the atomic-scale orderings. (a) Schematic figure of the projection for ABO_3 unit cell along the [110] zone axis. (b) Schematic illustration showing how to determine the position of each atom by simultaneously capturing ADF and ABF-iDPC images. (c) Schematic illustration of the anti-phase oxygen octahedron tilt. To have a better view about the oxygen octahedron, two oxygen atoms not appeared in the [100] projected direction are provided in dashed circles. (d) Schematic illustration of how to calculate the centroid difference between the cations and anions.

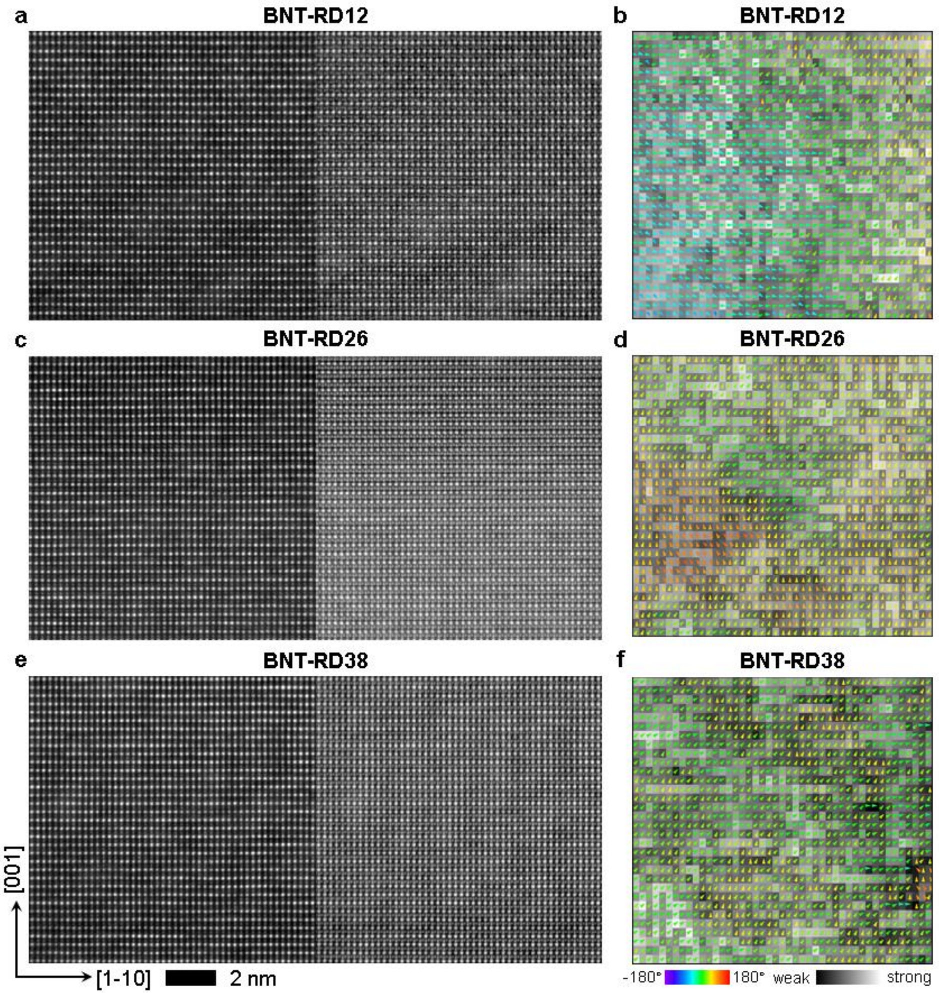

Supplementary Fig. 2 | Composition-dependent polarization mapping. Simultaneously captured (a, c, e) ADF and iDPC images and the corresponding (b, d, f) polarization mapping images for selected compositions of (a, b) BNT-RD12, (c, d) BNT-RD26 and (e, f) BNT-RD38. The left color bar shows the angle of polarization vectors, and the right color bar shows the magnitude of polarization vectors.

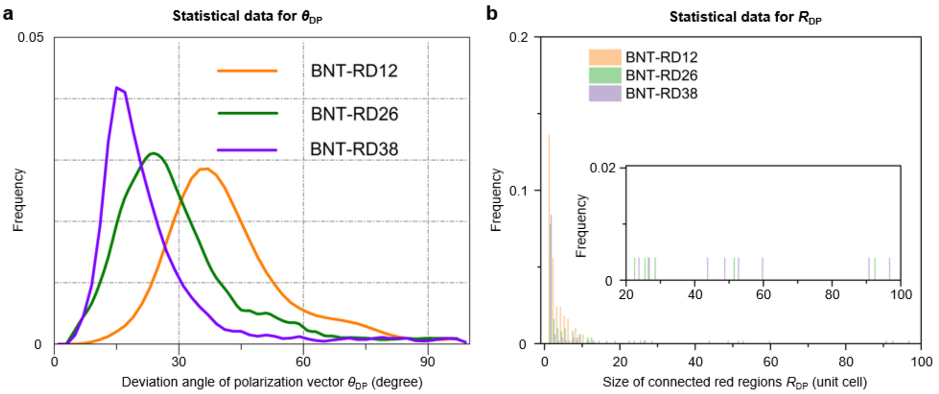

Supplementary Fig. 3 | Statistics of the composition-dependent deviated angle of polarization vectors. Statistical data for (a) the deviation angle of polarization vector θ_{DP} and (b) size of connected red regions R_{DP}.

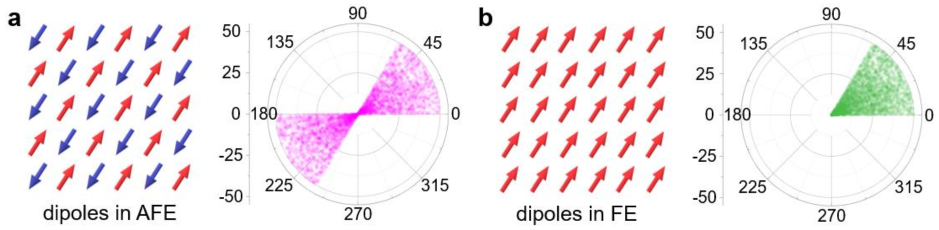

Supplementary Fig. 4 | Relative cation-anion displacement distribution for ideal AFE and FE orderings. Schematic illustration about the dipoles induced by the relative cation-anion displacement in ideal (a) AFE and (b) FE conditions.

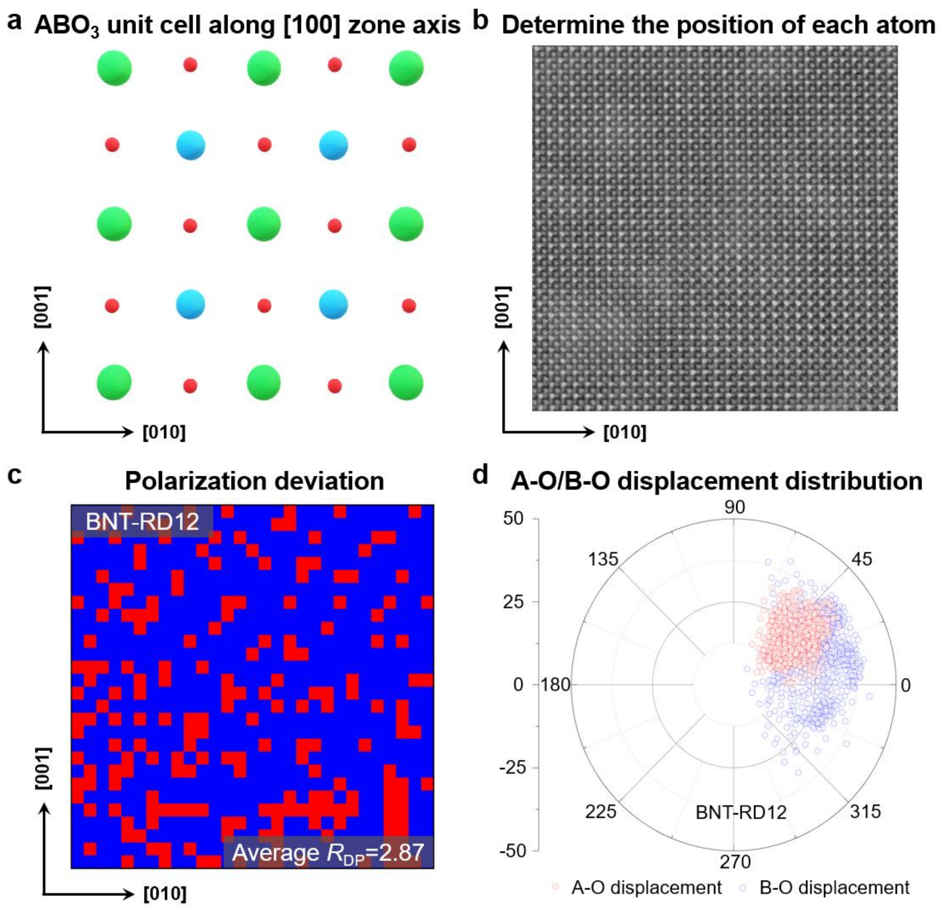

Supplementary Fig. 5 | Statistical data of polarization deviations along the [100] zone axis. (a) Schematic figure of the projected ABO_3 unit cell along the [100] zone axis. (b) Atomically resolved STEM image used to determine the position of each atom. (c) The $[100]_p$ projected local structure reflecting different deviation degrees of polarization angles in the BNT-RD12, using the same evaluation principle in Fig. 2b. (d) Compilation of the relative A-O/B-O displacement data in polar plots, which delineate the orientation and magnitude of displacement between cations and anions.

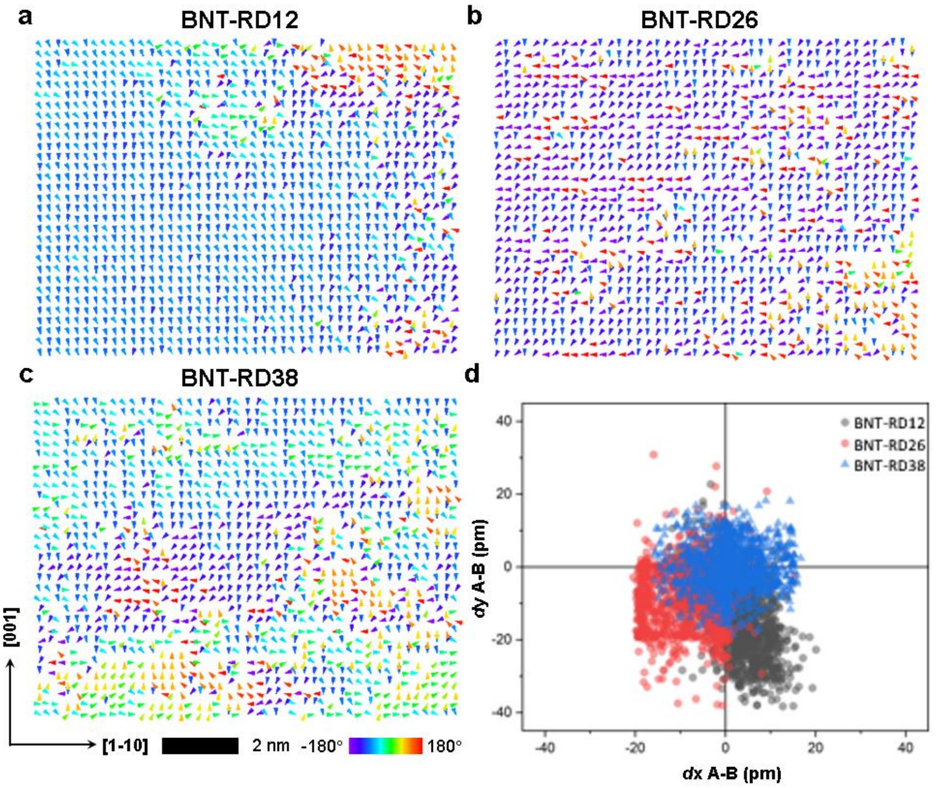

Supplementary Fig. 6 | Composition-dependent evolution of relative A-B displacement. Relative A-B displacement of (a) BNT-RD12, (b) BNT-RD26, and (c) BNT-RD38 compositions. (d) Data comparison of relative A-B displacement in BNT-RD12, BNT-RD26, and BNT-RD38 compositions.

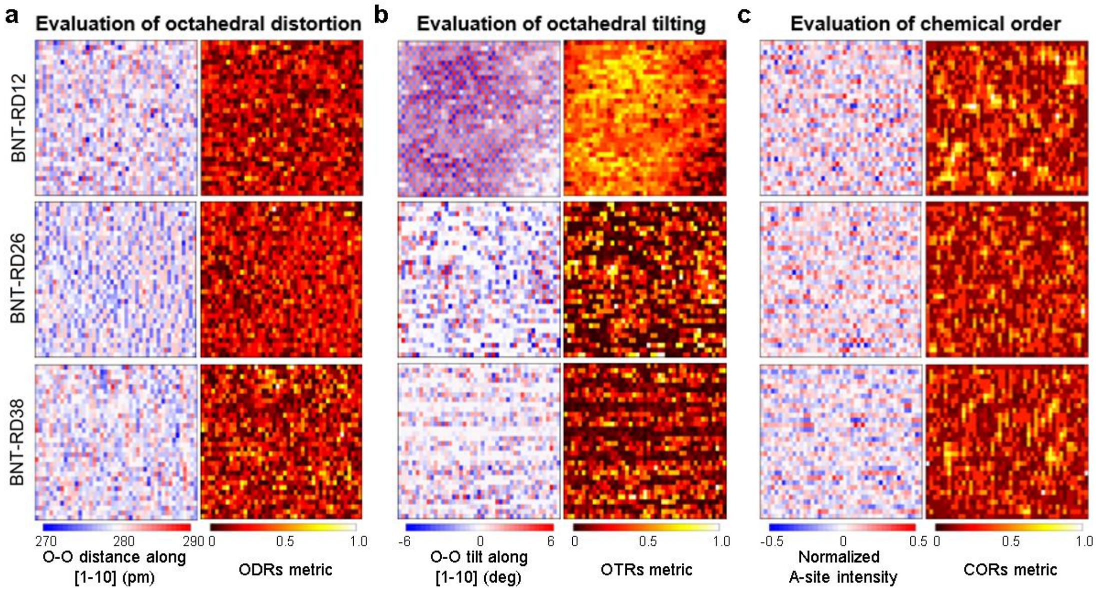

Supplementary Fig. 7 | Local chemical and structural heterogeneities mapping. This picture shows the statistical distribution of structural and chemical heterogeneities in different BNT-based compositions, including (a) octahedral distortion, (b) octahedral tilting, and (c) chemical order.

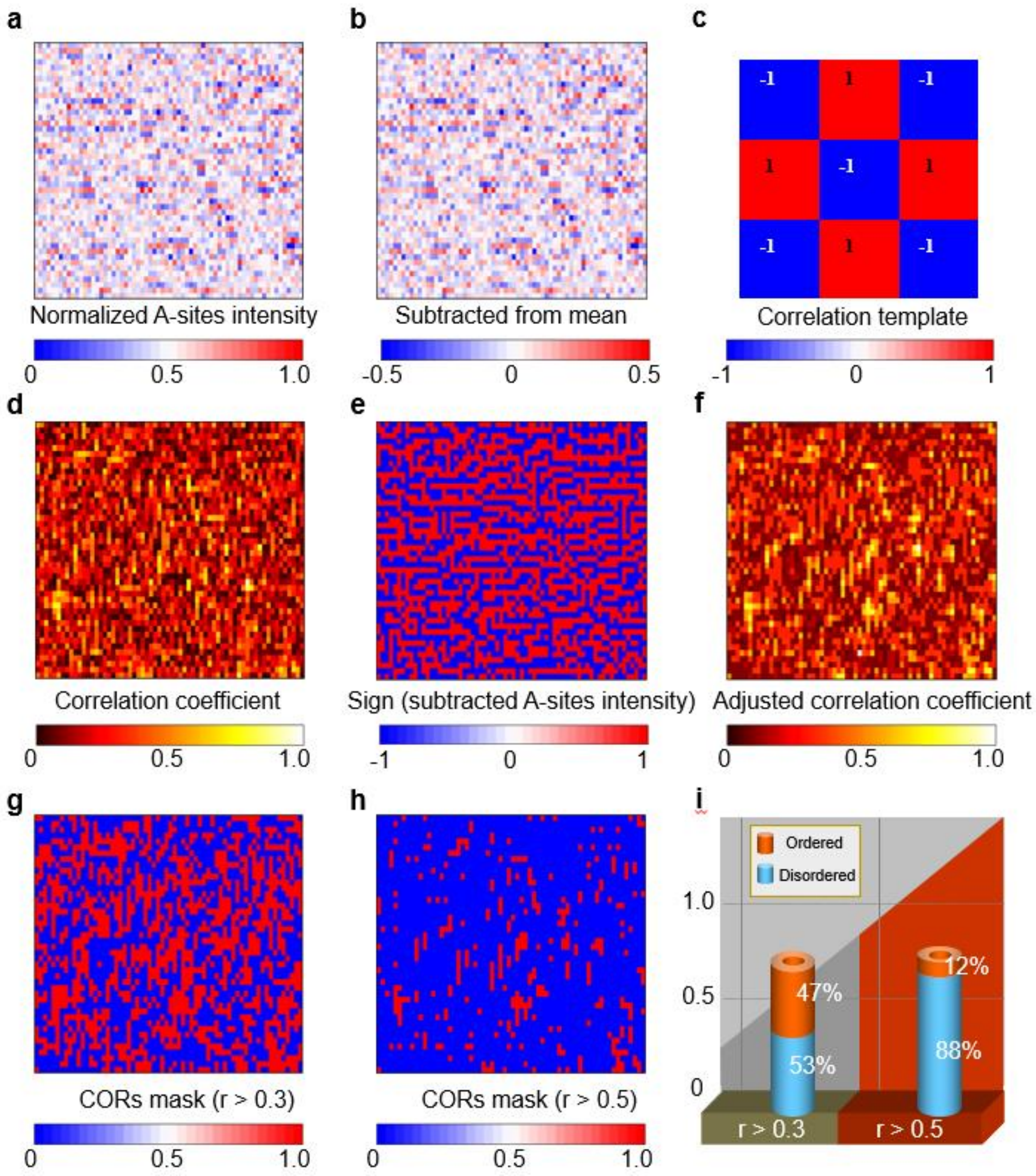

Supplementary Fig. 8 | Principles used to determine the local chemical orderings. (a) Normalized intensity data of A-site columns according to the average intensity of local 3×3 unit cells. (b) Subtracted intensity data of A-site columns from the local mean[2,4]. (c) Standard 3×3 correlation template used to evaluate the chemical ordering of A-site cations. (d) Calculated chemical ordering data. (e) Classified chemical ordering data by considering the negative intensity data as "-1" and positive intensity data as "+1". (f) Adjusted chemical ordering data. (g-i) Quantified chemical ordered regions after applying a threshold mask on the calculated data.

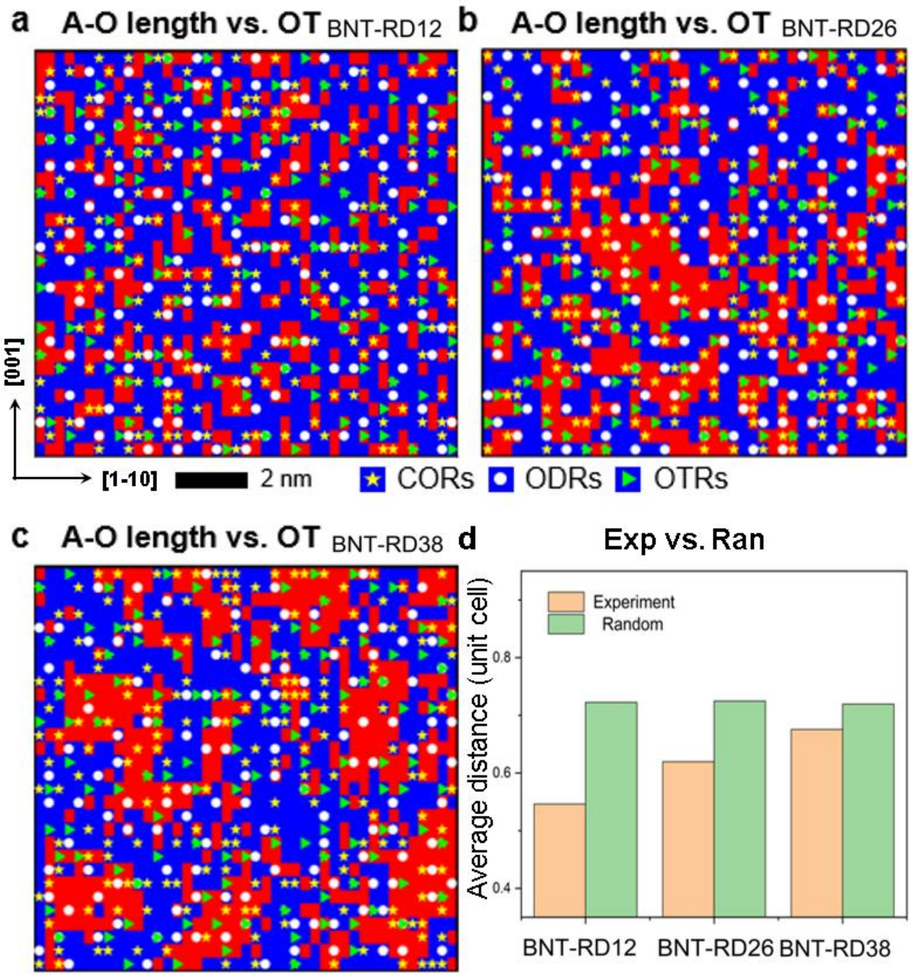

Supplementary Fig. 9 | Locate the positions of COR, ODR, OTR and spatially correlate them with the deviated polarizations. (a-c) The local maxima are used as the positions of these local ordering, and the filtered local polar structures are used as the background. (d) The distance between experimental homogeneities and red regions are evaluated by comparing the distance between randomly distributed homogeneities and red regions. The lower experimental values indicate that the local homogeneities are spatially correlated with the red regions.

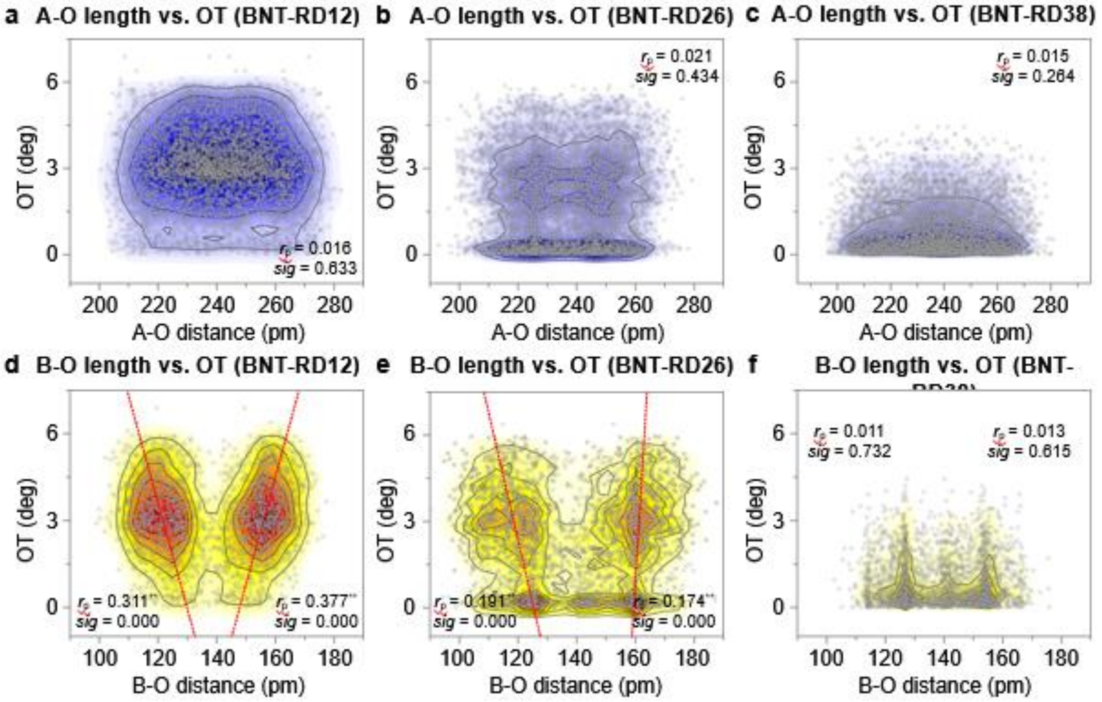

Supplementary Fig. 10 | Correlation analysis: A-O/B-O bond length vs. oxygen octahedral tilting. Direct point-to-point correlation analysis on the relationship between A-O/B-O bond length and the degree of oxygen octahedral tilting. r_p is the Pearson's correlation coefficient, ** indicates the significant correlation between two variables, sig is the significance.

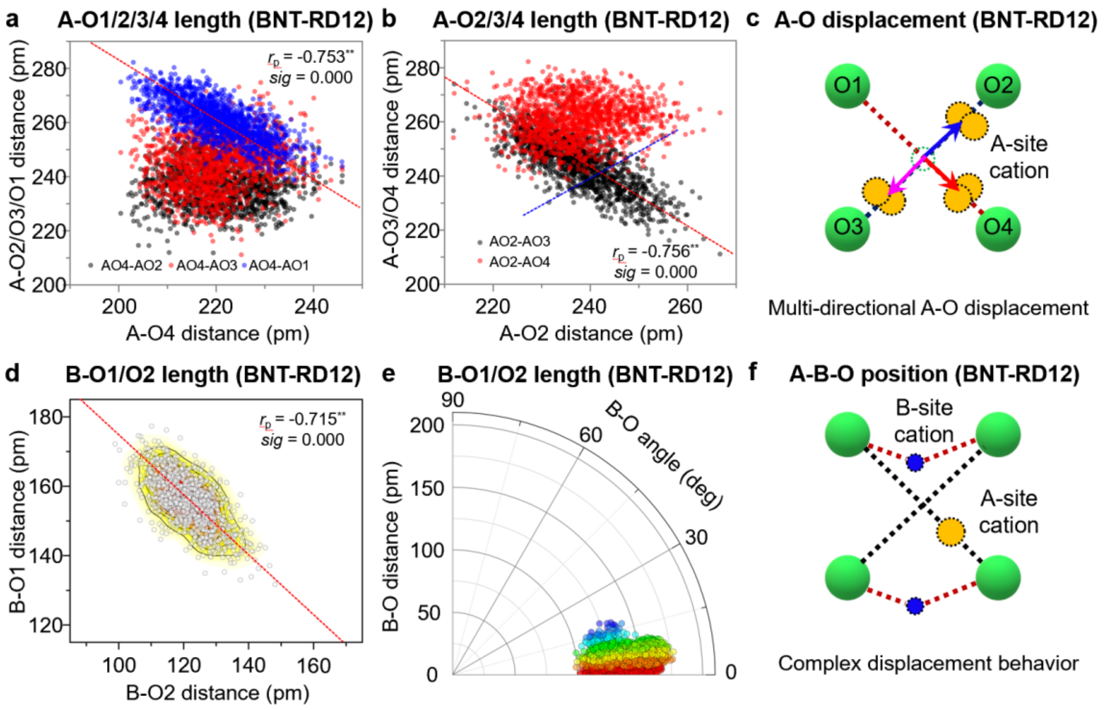

Supplementary Fig. 11 | Analysis of A-O and B-O bond length for BNT-RD12. The scatter data shown in (a, b, d, e) were obtained from STEM results, and (c, f) show the schematic illustration of relative positions of A-site, B-site cations, and O-site anions.

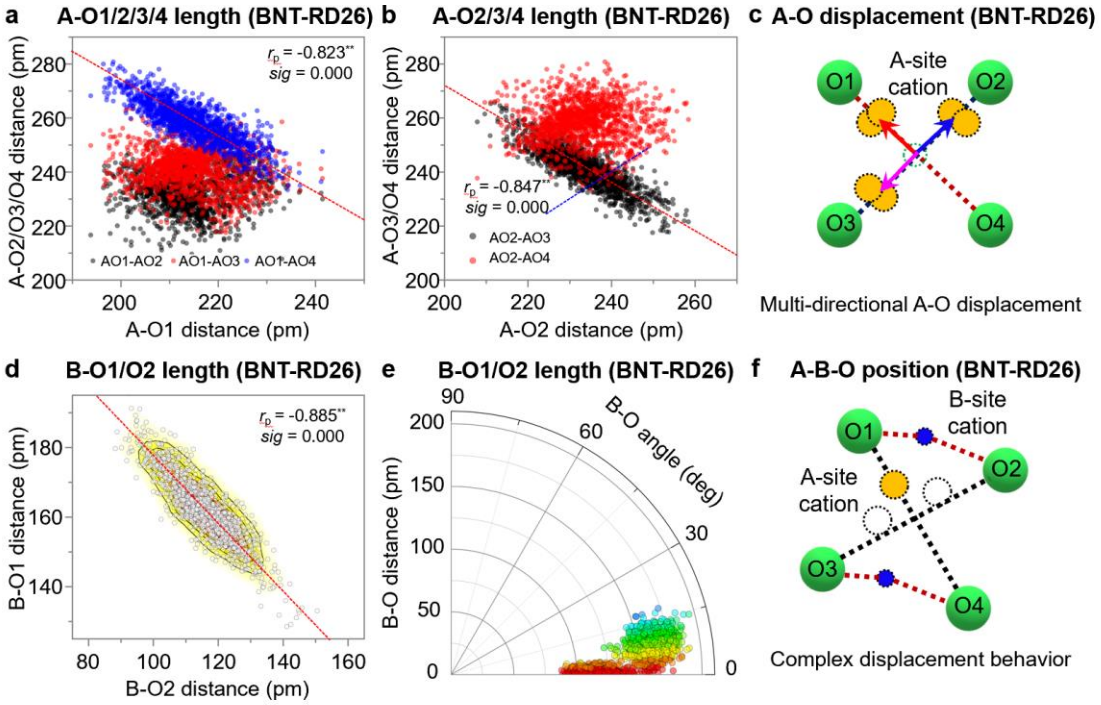

Supplementary Fig. 12 | Analysis of A-O and B-O bond length for BNT-RD26. The scatter data shown in (a, b, d, e) were obtained from STEM results, and (c, f) show the schematic illustration of relative positions of A-site, B-site cations, and O-site anions.

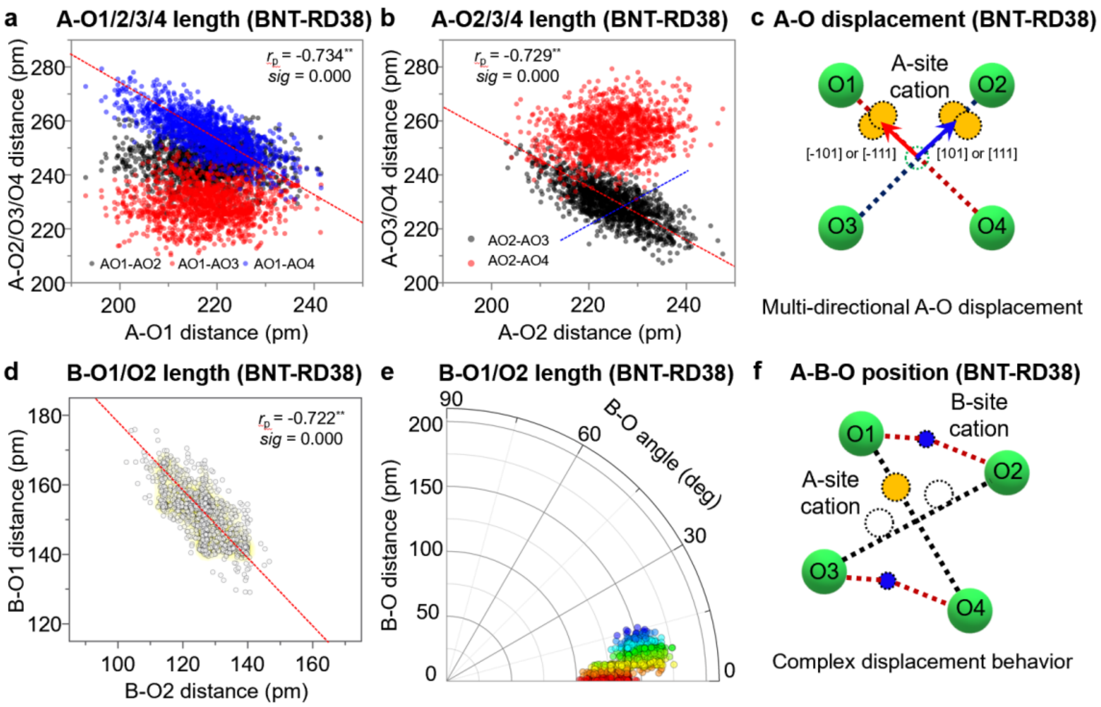

Supplementary Fig. 13 | Analysis of A-O and B-O bond length for BNT-RD38. The scatter data shown in (a, b, d, e) were obtained from STEM results, and (c, f) show the schematic illustration of relative positions of A-site, B-site cations, and O-site anions.

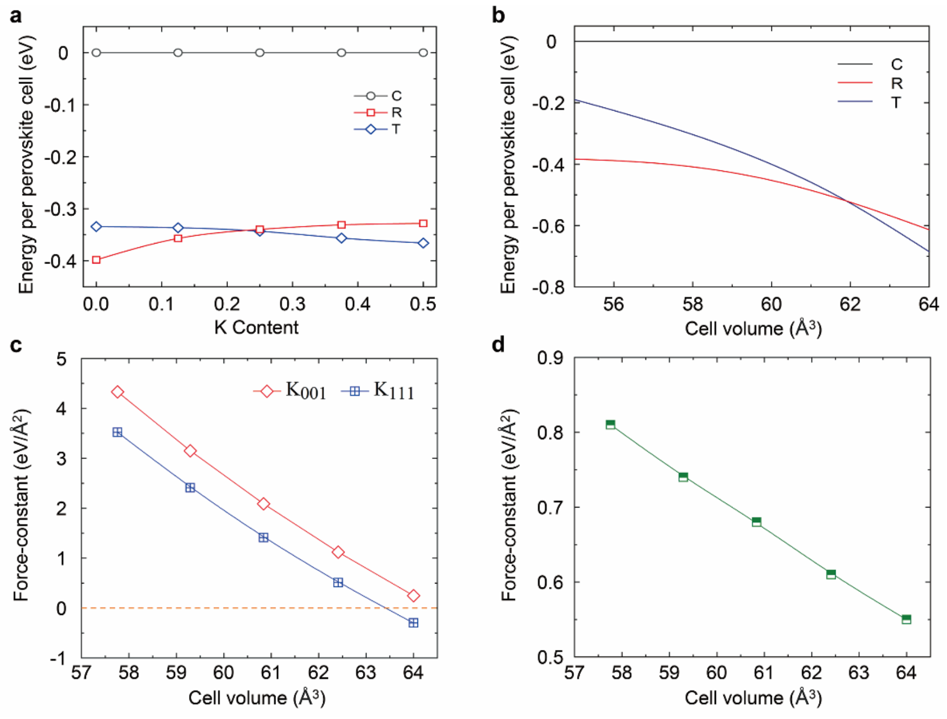

Supplementary Fig. 14 | First-principles calculation: driving force for the structural evolution in BNT. (a) The energy evolution of different symmetries with the increased content of K. The calculation result is obtained by applying the ordered A-site cations (Bi-K/Na-Bi-K/Na… order along [111] direction), where Bi atoms are fixed, and Na atoms are gradually replaced by K atoms. There is a phase transition from *R* to *T* as the K content is increased. (b) The energy evolution of different symmetries with the increased cell volume for the pure BNT. (c) The force constants of Ti in [001] and [111] directions, and (d) the corresponding difference of force-constant ($K_{001} - K_{111}$) against the lattice volume in pure BNT. The larger the force constant, the larger the resilience force when the ions displace. The low or even negative force constant is beneficial to reduce the energy of the system when displacement occurs. The smaller the lattice volume, the greater the difference of force-constants. That is, dipoles will displace towards [111] direction at the condition of smaller lattice volume.

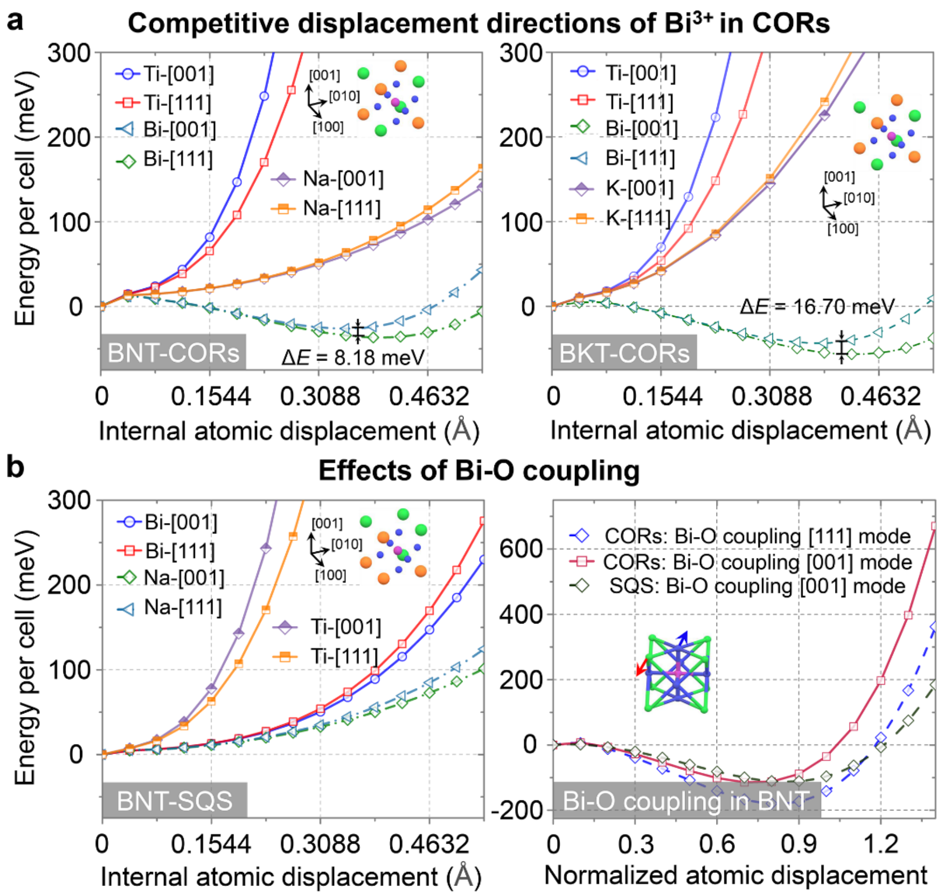

Supplementary Fig. 15 | First-principles calculations: multi-directional displacement of A-site cations. (a) The displacement directions of Bi^{3+} in BNT and BKT and (b) effects of Bi-O coupling in pure BNT cells. The ordered distributed Bi^{3+}/Na^{+} along [111] direction (CORs) is considered to simulate the chemical ordered condition, while the randomly distributed Bi^{3+}/Na^{+} along [111] direction (SQS) is considered to simulate the chemical disordered condition.

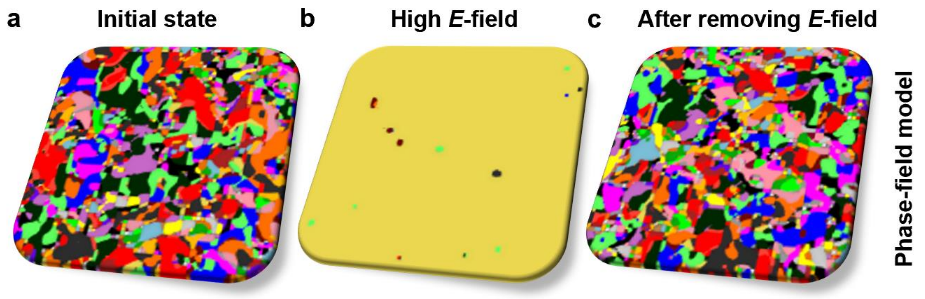

Supplementary Fig. 16 | *E*-field-dependent evolution of domain structures for BNT-RD38. The *E*-field-dependent domain evolutions are presented by phase-field simulation.

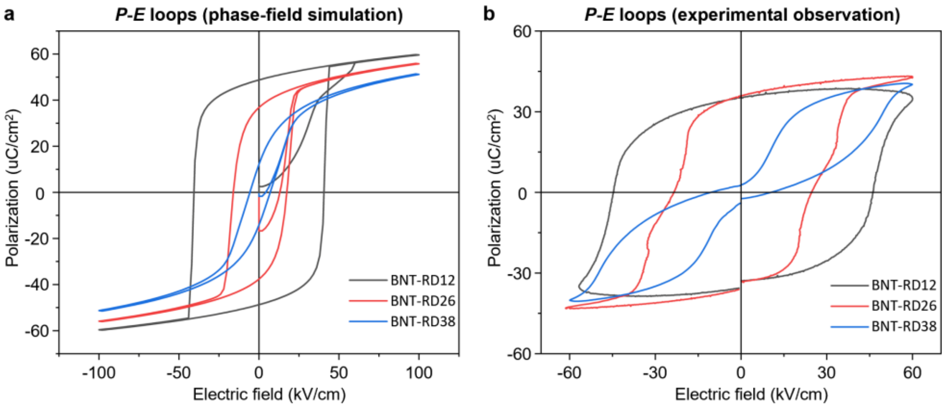

Supplementary Fig. 17 | Dynamic ferroelectric response: theoretical vs. experimental.

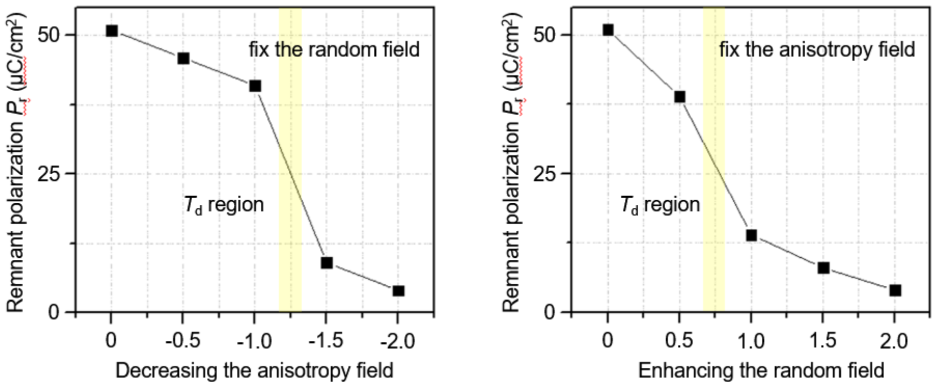

Supplementary Fig. 18 | Effects of the anisotropy field and random field on T_d. This figure shows that decreasing the anisotropy field and enhancing the random field will both induce the depolarization phenomenon. The definition of T_d is defined as "the temperature where the remanent polarization sharply reduces", according to the European standard on piezoelectric properties[23]. From this plot, we can conclude that reducing the anisotropy field and/or enhancing the random field will lead to the depolarization phenomenon (the remanent polarization sharply reduces to a low level).

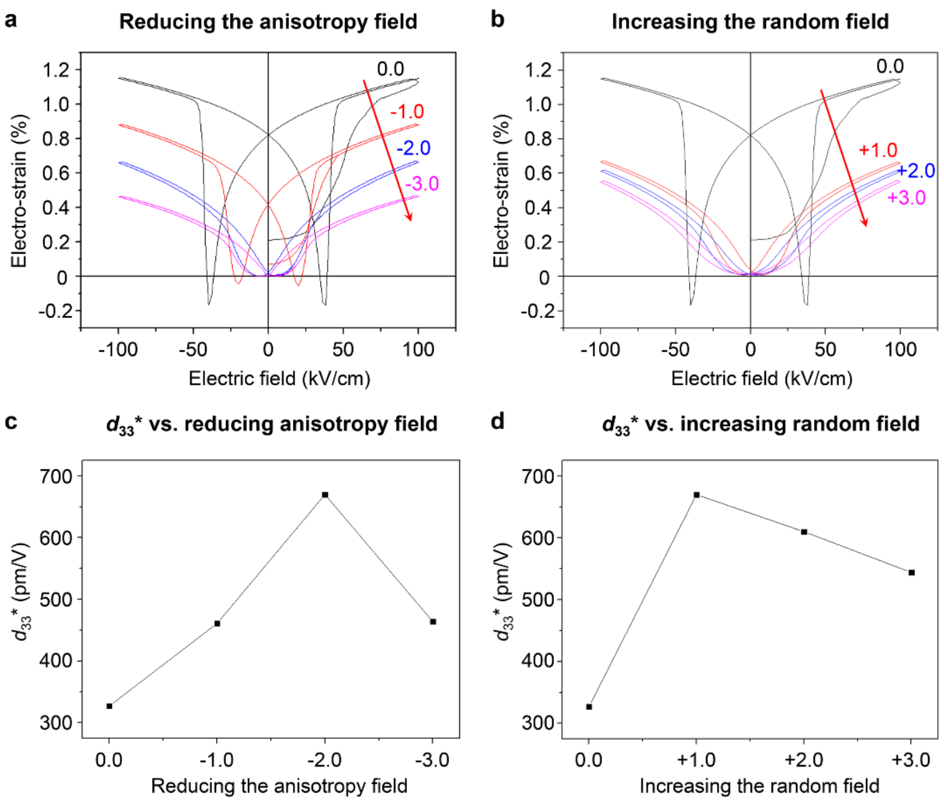

Supplementary Fig. 19 | The optimized d_{33}* is attributed to the competitive and synergetic equilibrium between anisotropy field and random field. These results are calculated by the phase-field model. d_{33}* increases first, and then decreases.

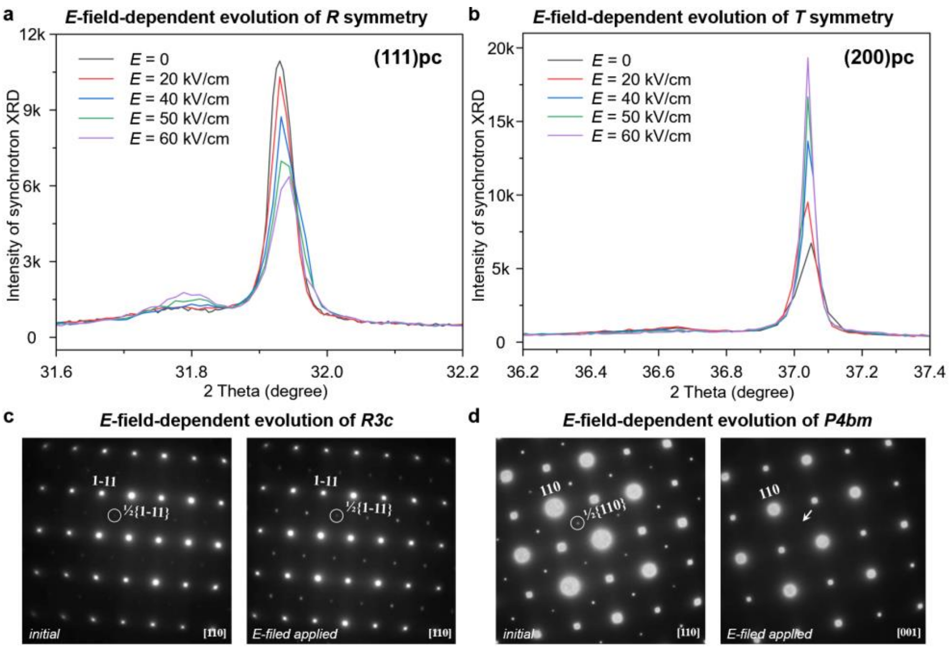

Supplementary Fig. 20 | *E*-field-dependent evolution of average and local symmetries for BNT-RD38. (a, b) The *E*-field-dependent evolution of average phase structures was presented according to the characteristic peaks of {111}pc and {200}pc, including the (a) rhombohedral symmetry and (b) tetragonal symmetry. (c, d) The *E*-field-dependent evolution of local symmetries was presented according to the superlattice diffraction spots, including the (c) 1/2{*ooo*} for rhombohedral symmetry and (d) 1/2{*ooe*} for tetragonal symmetry (*o* and *e* represent odd and even Miller indices).

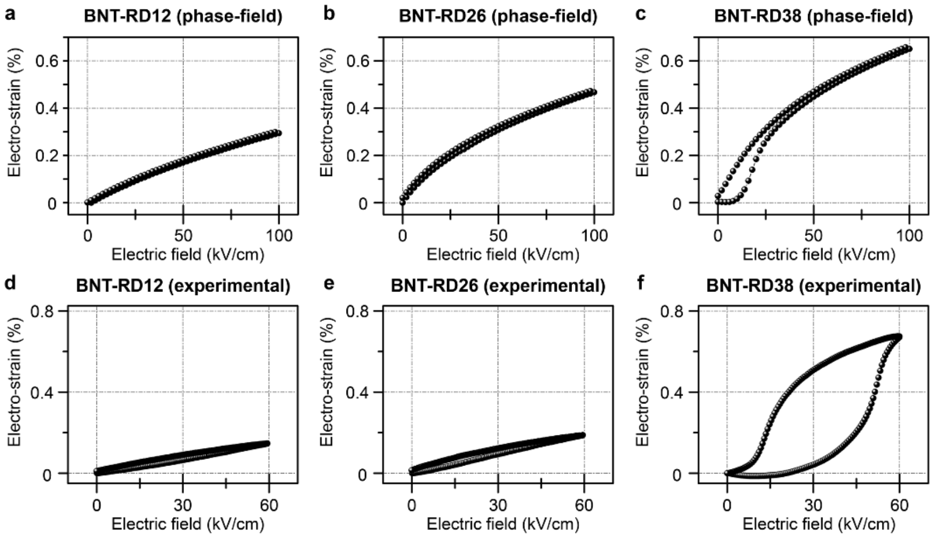

Supplementary Fig. 21 | Dynamic electromechanical response: phase-field simulation and experimental results.

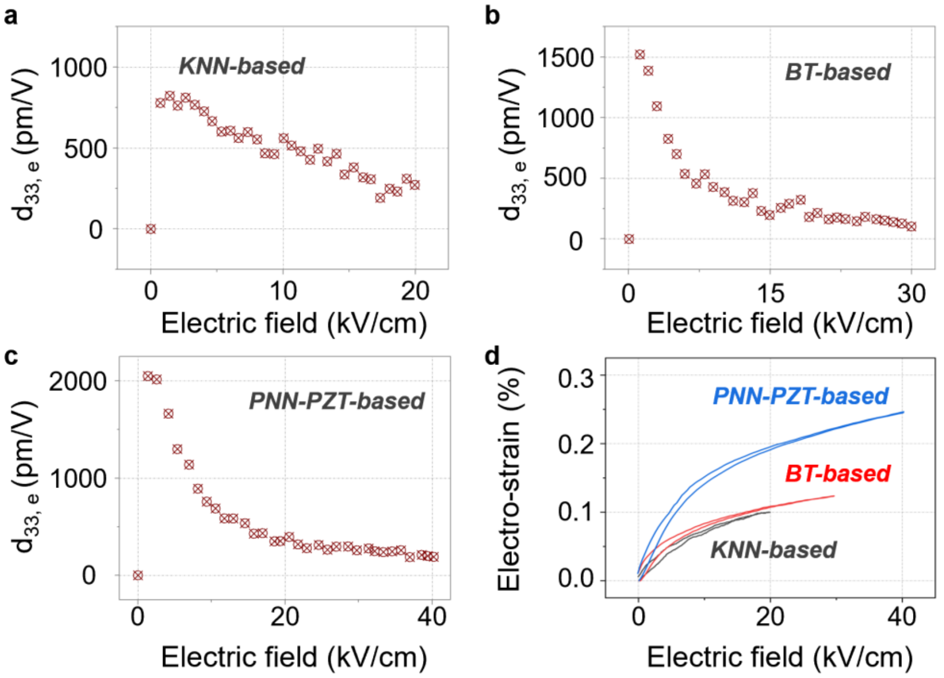

Supplementary Fig. 22 | Dynamic electromechanical response in representative piezoelectric ceramic systems. The selected material systems are the piezoelectric ceramics that exhibit the state-of-the-art small-signal piezoelectricity, including (a) KNN-based ceramic sample with the composition of 0.944 $K_{0.48}Na_{0.52}Nb_{0.95}Sb_{0.05}O_3$ – 0.04 $Bi_{0.5}(Na_{0.82}K_{0.18})_{0.5}ZrO_3$ – 0.4% Fe_2O_3 – 1.6%$AgSbO_3$[24], (b) BT-based ceramic sample 0.82 $Ba(Ti_{0.89}Sn_{0.11})O_3$ – 0.18 $(Ba_{0.7}Ca_{0.3})TiO_3$[25], and (c) PZT-based ceramic sample with the composition of 0.55 $Pb(Ni_{1/3}Nb_{2/3})O_3$ - 0.45 $Pb(Zr_{0.3}Ti_{0.7})O_3$ – 0.5wt%Ta_2O_5[26]. Samples used for the measurement were provided by the authors in references [24-26]. For these representative ceramic systems, one can see that their dynamic electromechanical responses reach the maximum at the low electric field condition, which is obviously different from that in BNT-based ceramic system.

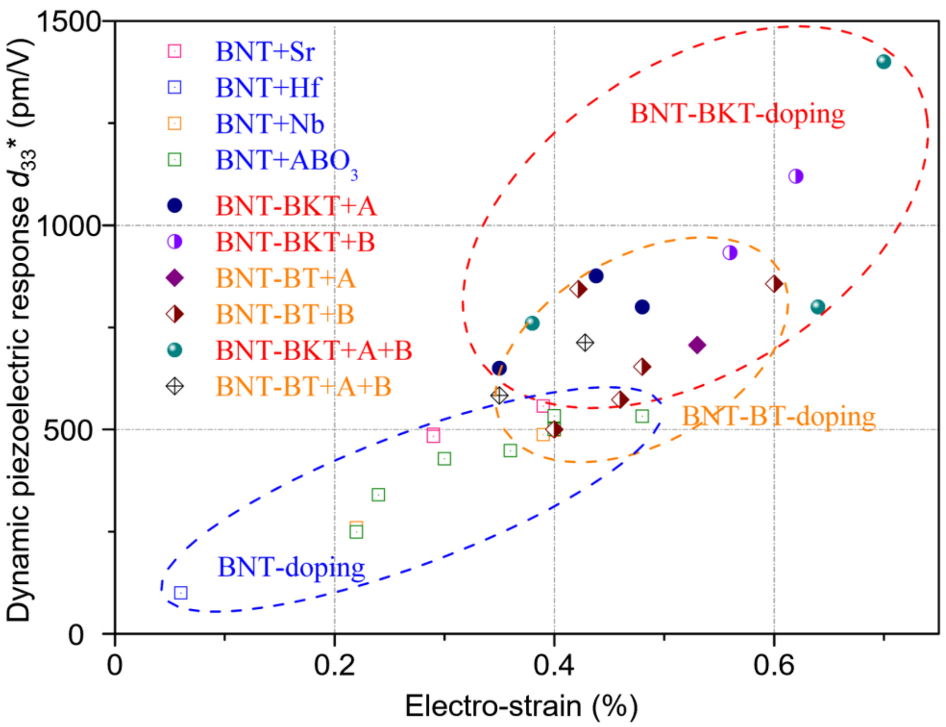

Supplementary Fig. 23 | Effects of chemical modification on the dynamic electromechanical response. The data are obtained from our experimental results and reported literature[27-41]. Compared with the results of chemical doping on BNT and BNT-BT materials, chemical doping on the BNT-BKT exhibits the better dynamic piezoelectric performance. From this figure, three general observations can be summarized: (1) The BNT-based material with higher piezoelectric charge coefficient d_{33} is easier to achieve a larger piezoelectric strain coefficient d_{33}^* by doping [*e.g.*, in contrast to BNT, the larger d_{33}^* values can be obtained in BNT-BT and BNT-BKT]; (2) Heterovalent cation doping is more effective in decreasing T_d and enhancing d_{33}^* than isovalent cation doping [*e.g.*, heterovalent Nb^{5+}/Ta^{5+} are more effective to decrease T_d and enhance d_{33}^* than isovalent Hf^{4+}/Zr^{4+}]; (3) In contrast to A-site doping, B-site doping requires a much lower doping concentration to shift T_d to the ambient temperature [*e.g.*, to decrease T_d to the ambient temperature and enhance d_{33}^*, doping concentration of > 20% is required for A-site, while doping concentration of < 5% is required for B-site].

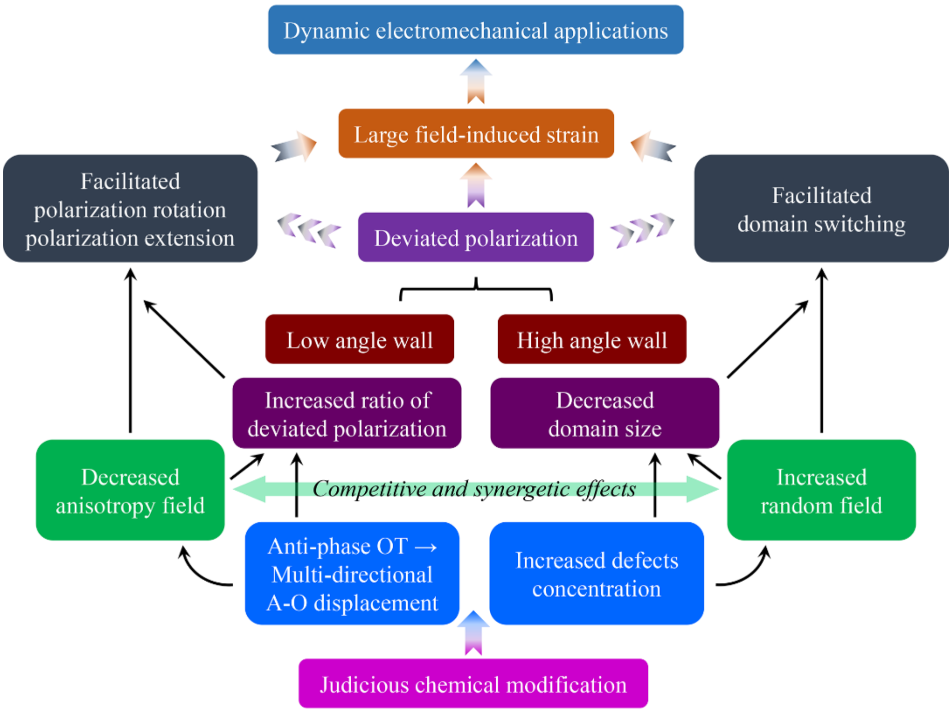

Supplementary Fig. 24 | The overall framework of this work. The composition-structure-performance correlation and the underlying mechanisms are built up, as shown in this picture. Facilitated polarization rotation, polarization extension and domain switching processes account for the giant transient thrust and the large dynamic electromechanical performance, originating from two kinds of deviated polarizations. Through the judicious chemical modification, the increased defect-induced built-in random field is the dominant driving force for deviating polarizations with high-angle walls, leading to the increased density of high-angle domain walls and decreased domain size. Meanwhile, the dominant driving force for deviating polarizations with low-angle walls changes from the anti-phase oxygen octahedral tilting to the multidirectional A-O displacements, leading to the decreased anisotropy field and the increased ratio of deviated polarizations with low-angle walls. From the phenomenological perspective, the optimized dynamic electromechanical performance is the result of a competitive and synergetic equilibrium between anisotropy field and random field.

Supplementary Table 1. The correlation data shown in Figs. 3d-3i.

	BNT-RD12		BNT-RD26		BNT-RD38	
	r_p	sig	r_p	sig	r_p	sig
**DP-OT**	0.268**	0.000	0.305**	0.000	0.027	0.383
**DP-OD**	0.030	0.443	0.038	0.153	0.021	0.481
**DP-r_{CO}**	0.014	0.785	0.026	0.315	-0.053	0.124
**d_{AO}-I_A**	-0.194**	0.000	-0.219**	0.000	-0.353**	0.000
**$d_{AO(+)}$-I_A**	0.134**	0.000	0.286**	0.000	0.390**	0.000
**$d_{AO(-)}$-I_A**	0.991**	0.000	-0.151**	0.000	-0.288**	0.000

r_p is the Pearson's correlation coefficient, ** indicates the significant correlation between two variables, and sig is the significance factor.

Supplementary References

1 de Graaf, S., Momand, J., Mitterbauer, C., Lazar, S. & Kooi, B. J. Resolving hydrogen atoms at metal-metal hydride interfaces. *Science Advances* **6**, eaay4312 (2020).

2 Kumar, A. *et al.* Atomic-resolution electron microscopy of nanoscale local structure in lead-based relaxor ferroelectrics. *Nature Materials* **20**, 62-67 (2021).

3 Glazer, A. M. J. A. C. S. B. S. C. & Chemistry, C. The classification of tilted octahedra in perovskites. *Acta Crystallographica Section B: Structural Crystallography and Crystal Chemistry* **28**, 3384-3392 (1972).

4 Abrahams, S., Kurtz, S. & Jamieson, P. J. P. R. Atomic displacement relationship to Curie temperature and spontaneous polarization in displacive ferroelectrics. *Physical Review* **172**, 551 (1968).

5 Jia, C.-L. *et al.* Unit-cell scale mapping of ferroelectricity and tetragonality in epitaxial ultrathin ferroelectric films. *Nature Materials* **6**, 64-69 (2007).

6 Das, S. *et al.* Observation of room-temperature polar skyrmions. *Nature* **568**, 368-372 (2019).

7 Jia, C.-L., Urban, K. W., Alexe, M., Hesse, D. & Vrejoiu, I. J. S. Direct observation of continuous electric dipole rotation in flux-closure domains in ferroelectric $Pb(Zr,Ti)O_3$. *Science* **331**, 1420-1423 (2011).

8 Jia, C.-L. *et al.* Nanodomains and nanometer-scale disorder in multiferroic bismuth ferrite single crystals. *Acta Materialia* **82**, 356-368 (2015).

9 Lee, D. *et al.* Emergence of room-temperature ferroelectricity at reduced dimensions. *Science* **349**, 1314-1317 (2015).

10 Polking, M. J. *et al.* Ferroelectric order in individual nanometre-scale crystals. *Nature Materials* **11**, 700-709 (2012).

11 Tao, H. *et al.* Ultrahigh performance in lead-free piezoceramics utilizing a relaxor slush polar state with multiphase coexistence. *Journal of the American Chemical Society* **141**, 13987-13994 (2019).

12 Peters, J. J., Apachitei, G., Beanland, R., Alexe, M. & Sanchez, A. M. J. N. c. Polarization curling and flux closures in multiferroic tunnel junctions. *Nature Communications* **7**, 1-7 (2016).

13 Kresse, G. & Hafner, J. Ab initio molecular-dynamics simulation of the liquid-metal–amorphous-semiconductor transition in germanium. *Physical Review B* **49**, 14251 (1994).

14 Kresse, G. & Furthmüller, J. Efficient iterative schemes for ab initio total-energy calculations using a plane-wave basis set. *Physical Review B* **54**, 11169 (1996).

15 Tan, Z., Xing, J., Peng, Y., Zhang, Q. & Zhu, J. Polarization rotation boosts strong piezoelectric response in the lead-free perovskite ferroelectric $K_{0.5}Na_{0.5}NbO_3$. *Physical Review B* **104**, 014104 (2021).

16 Perdew, J. P. *et al.* Restoring the density-gradient expansion for exchange in solids and surfaces. *Physical Review Letters* **100**, 136406 (2008).

17 Blöchl, P. E. Projector augmented-wave method. *Physical Review B* **50**, 17953 (1994).

18 Van de Walle, A. *et al.* Efficient stochastic generation of special quasirandom structures. *Calphad* **42**, 13-18 (2013).

19 Kholkin, A. *et al.* Surface domain structures and mesoscopic phase transition in relaxor ferroelectrics. *Advanced Functional Materials* **21**, 1977-1987 (2011).

20 Gao, R., Shi, X., Wang, J., Zhang, G. & Huang, H. Designed giant room-temperature electrocaloric effects in metal-free organic perovskite [MDABCO](NH4)I_3 by phase–field simulations. *Advanced Functional Materials* **31**, 2104393 (2021).

21 Zhang, L. *et al.* A new strategy for large dynamic piezoelectric responses in lead-free ferroelectrics: the relaxor/morphotropic phase boundary crossover. *Advanced Functional Materials* **30**, 2004641 (2020).

22 Niu, C. *et al.* Spin-driven ordering of Cr in the equiatomic high entropy alloy NiFeCrCo. *Applied Physics Letters* **106**, 161906 (2015).

23 Anton, E. M. *et al.* Determination of depolarization temperature of $(Bi_{1/2}Na_{1/2})TiO_3$-based lead-free piezoceramics. *Journal of Applied Physics* **110**, 094108 (2011).

24 Tao, H. *et al.* Ultrahigh performance in lead-free piezoceramics utilizing a relaxor slush polar

state with multiphase coexistence. *Journal of the American Chemical Society* **141**, 13987-13994 (2019).

25 Zhao, C. *et al.* Practical high piezoelectricity in barium titanate ceramics utilizing multiphase convergence with broad structural flexibility. *Journal of the American Chemical Society* **140**, 15252-15260 (2018).

26 Wang, H. *et al.* Giant piezoelectric coefficient of PNN-PZT-based relaxor piezoelectric ceramics by constructing an RT MPB. *Ceramics International* **47**, 12284-12291 (2021).

27 Hiruma, Y., Imai, Y., Watanabe, Y., Nagata, H. & Takenaka, T. J. A. P. L. Large electrostrain near the phase transition temperature of $(Bi_{0.5}Na_{0.5})TiO_3$–$SrTiO_3$ ferroelectric ceramics. *Applied Physics Letters* **92**, 262904 (2008).

28 Zuo, R., Wang, H., Ma, B. & Li, L. J. J. o. M. S. M. i. E. Effects of Nb^{5+} doping on sintering and electrical properties of lead-free $(Bi_{0.5}Na_{0.5})TiO_3$ ceramics. *Journal of Materials Science: Materials in Electronics* **20**, 1140-1143 (2009).

29 Liu, X. & Tan, X. Giant strains in non-textured $(Bi_{1/2}Na_{1/2})TiO_3$-based lead-free ceramics. *Advanced Materials* **28**, 574-578 (2016).

30 Liu, X. & Tan, X. Giant strain with low cycling degradation in Ta-doped $[Bi_{1/2}(Na_{0.8}K_{0.2})_{1/2}]TiO_3$ lead-free ceramics. *Journal of Applied Physics* **120**, 034102 (2016).

31 Zhang, Y., Zhao, C., Yin, J. & Wu, J. Effect of Hf and Li on the structure and electrical properties of $Bi_{0.5}Na_{0.5}TiO_3$ lead-free ceramics. *Journal of Materials Science: Materials in Electronics* **28**, 16948-16954 (2017).

32 Yin, J., Zhao, C., Zhang, Y. & Wu, J. Ultrahigh strain in site engineering-independent $Bi_{0.5}Na_{0.5}TiO_3$-based relaxor-ferroelectrics. *Acta Materialia* **147**, 70-77 (2018).

33 Zuo, R., Wang, H., Ma, B. & Li, L. Effects of Nb^{5+} doping on sintering and electrical properties of lead-free $(Bi_{0.5}Na_{0.5})TiO_3$ ceramics. *Journal of Materials Science: Materials in Electronics* **20**, 1140 (2009).

34 Chen, J., Wang, Y., Zhang, Y., Yang, Y. & Jin, R. Giant electric field-induced strain at room temperature in $LiNbO_3$-doped $0.94(Bi_{0.5}Na_{0.5})TiO_3$-$0.06BaTiO_3$. *Journal of the European Ceramic Society* **37**, 2365-2371 (2017).

35 Jiao, L. *et al.* Morphotropic phase boundary and electric properties in $(1-x)Bi_{0.5}Na_{0.5}TiO_3$–$xBaSnO_3$ lead-free piezoelectric ceramics. *Journal of Materials Science: Materials in Electronics* **24**, 4080-4084 (2013).

36 Bai, W. *et al.* Structure and electromechanical properties in $Bi_{0.5}Na_{0.5}TiO_3$-based lead-free piezoceramics with calculated end-member $Bi(Ni_{0.5}Ti_{0.5})O_3$. *Journal of the European Ceramic Society* **35**, 3457-3466 (2015).

37 Bai, W., Bian, Y., Hao, J., Shen, B. & Zhai, J. The Composition and Temperature-Dependent Structure Evolution and Large Strain Response in $(1-x)(Bi_{0.5}Na_{0.5})TiO_3$–$xBa(Al_{0.5}Ta_{0.5})O_3$ Ceramics. *Journal of the American Ceramic Society* **96**, 246-252 (2013).

38 Maqbool, A. *et al.* Ferroelectric and piezoelectric properties of $SrZrO_3$-modified $Bi_{0.5}Na_{0.5}TiO_3$ lead-free ceramics. *Transactions of Nonferrous Metals Society of China* **24**, s146-s151 (2014).

39 Li, F., Zuo, R., Zheng, D. & Li, L. Phase-composition-dependent piezoelectric and electromechanical strain properties in $(Bi_{1/2}Na_{1/2})TiO_3$–$Ba(Ni_{1/2}Nb_{1/2})O_3$ lead-free ceramics. *Journal of the American Ceramic Society* **98**, 811-818 (2015).

40 Hiruma, Y., Nagata, H. & Takenaka, T. Detection of morphotropic phase boundary of $(Bi_{1/2}Na_{1/2})TiO_3$–$Ba(Al_{1/2}Sb_{1/2})O_3$ solid-solution ceramics. *Applied Physics Letters* **95**, 052903 (2009).

41 Hiruma, Y., Nagata, H. & Takenaka, T. Formation of morphotropic phase boundary and electrical properties of $(Bi_{1/2}Na_{1/2})TiO_3$–$Ba(Al_{1/2}Nb_{1/2})O_3$ solid solution ceramics. *Japanese Journal of Applied Physics* **48**, 09KC08 (2009).
